# *Operando* benchtop NMR study of ion transport through fluorine-free polymer membranes in a symmetric redox flow cell

**DOI:** 10.1039/d5ta06160a

**Published:** 2026-02-02

**Authors:** Giu A. Silva Testa, Marta Santos Rodríguez, Juan Carlos Martínez-López, Ángel E. Lozano, Cristina Álvarez, Javier Carretero-González, Evan Wenbo Zhao

**Affiliations:** a Magnetic Resonance Research Center, Institute for Molecules and Materials, Radboud University Heyendaalseweg 135 6525 AJ Nijmegen The Netherlands evanwenbo.zhao@ru.nl; b Institute of Polymer Science and Technology, ICTP, CSIC C/Juan de la Cierva, 3 Madrid 28006 Spain jcarretero@ictp.csic.es cristina.alvarez@ictp.csic.es

## Abstract

Polymeric membranes play a key role in redox flow batteries, where they regulate ion transport and contribute to overall battery performance. Current benchmark membranes are usually perfluorinated, which increases cost and environmental impact. Here, we synthesized and tested biphenyl-isatin polymers as cation exchange membranes in a pH neutral iron-based symmetric redox flow cell. We examined the effect of sulfonation on membrane permselectivity by measuring the diffusion of common supporting electrolytes (LiCl, NaCl, KCl) and assessing crossover rejection of larger redox-active anions such as ferricyanide. The membrane with the highest performance was implemented in a ferro/ferricyanide-based symmetric redox flow cell, demonstrating 92% capacity retention over 180 cycles. These findings indicate that fluorine-free sulfonated polymers can serve as viable alternatives to perfluorinated membranes in electrochemical technologies. In parallel, we demonstrated an *operando* benchtop NMR method with atomic specificity for identifying and quantifying Li^+^ charge-balancing ions through the biphenyl-isatin-based membrane. The method involved addressing paramagnetic relaxation attenuation of ^7^Li NMR intensity by first quantifying ferricyanide ions with the Evans method, followed by applying relaxation correction and quantification of Li^+^ cations. We observed that at low current densities, Li^+^ ions served as the primary charge-balancing species, whereas at higher current, a deviation between charge and Li^+^ concentration emerged, suggesting additional contributions from other ionic species. The relaxation–correction protocol introduced here enables accurate quantification of ion transport in symmetric redox flow cells containing paramagnetic species such as ferricyanide and potentially many organic radicals. This approach provides a general framework for studying ion transport and guiding the design of next-generation membranes for diverse redox chemistries.

## Abstract 2

*Operando* ^7^Li-NMR reveals and quantifies charge-balancing ion transport through a fluorine-free biphenyl-isatin-based membrane in a symmetric iron-based redox flow cell.

## Graphical abstract

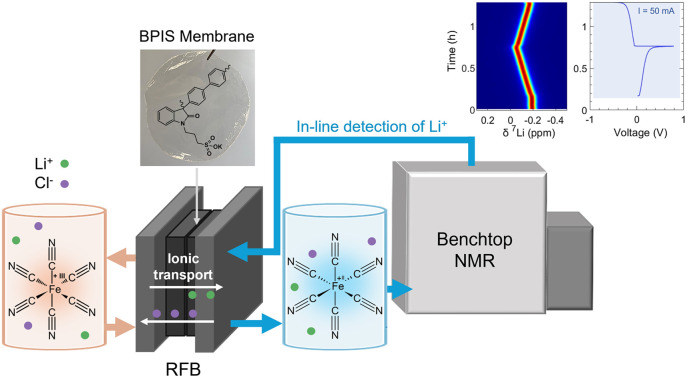

## Introduction

The growing integration of renewable energy sources like wind and solar has created an urgent demand for large-scale, flexible energy storage technologies capable of balancing intermittent generation with steady power supply. Among the various contenders, redox flow batteries (RFBs) have emerged as one of the most promising solutions, combining chemical tunability with engineering scalability. A RFB stores energy in two liquid electrolytes, known as the posolyte and negolyte, each containing redox active molecules that are stored in external tanks and circulated through an electrochemical cell during operation.^1–3^ During charge and discharge, these solutions undergo electrochemical reactions, converting electrical energy into chemical energy, and back to electrical energy, respectively. Because the energy capacity depends on the volume of the electrolyte, and the power output on the cell's design and kinetics, these two parameters can be tuned independently, which offers a unique advantage over other conventional batteries.^4^ This decoupling of energy and power makes RFBs ideally suitable for grid-scale storage, where long-duration discharge and flexible operation are essential characteristics. Their architecture also enables easy maintenance, long cycle life, and safe operation, especially when using aqueous electrolytes.^5^ However, the overall performance still hinges on efficient ion transport through the membrane, which must allow for charge-balancing ions to pass through while preventing cross-mixing of the active redox species.

Nafion membranes have become the benchmark in RFB research and applications due to their high ionic conductivity and exceptional chemical stability.^6^ Despite their widespread use, Nafion as a material faces important limitations related to both performance and cost.^7,8^ It offers limited selectivity and is expensive to produce, which constrains its practical and economic viability. Beyond these issues, Nafion is also a polyfluoroalkyl substance (PFAS), a class of compounds known for their environmental persistence and potential toxicity. Growing concern over PFAS has led to increasing regulatory scrutiny, particularly in Europe, where a comprehensive ban is currently under consideration.^9^ These pressures emphasize the urgency of developing alternative membrane materials that are both high-performing and environmentally benign. In addition to sustainability and regulatory compliance, identifying new polymers suitable for membrane application is crucial for advancing RFB technology. Critically, in the absence of viable alternatives, an effective ban of all PFAS could disrupt the broader deployment of clean energy systems,^10^ pushing the community to accelerate the development of safer, scalable alternatives for membrane applications. In this context, understanding the key performance requirements of next generation membranes is essential. Effective membrane design must enable high ionic transport for balancing charges while limiting crossover of redox-active species to maintain the system's efficiency and stability. In addition, membranes should exhibit strong chemical resistance, mechanical robustness, long-term durability, and cost-effectiveness.^11^ To meet all these criteria, the chemical composition and the structure of the membranes can be engineered by optimizing their polymer backbone, functional groups, and microphase morphology.^12^ Strategies such as incorporating hydrophilic domains through sulfonation, grafting ion-conducting side chains, or designing block copolymers with phase-separated architectures allow for controlled ion transport while restricting crossover.^12^ For instance, Nafion membranes feature per-fluorinated side chains terminated with sulfonic acid groups (–SO_3_H), which form interconnected hydrophilic domains. These well-organized networks enable exceptionally high proton conductivity, inspiring researchers to mimic this channel architecture.^13^ In recent years, hydrocarbon-based fluorine-free membranes have emerged as promising alternatives to Nafion,^14^ offering tunable properties suited to specific chemistries. Notably, high molecular-weight polymers synthesized through superacid-catalyzed polyhydroxylakylation of aromatic hydrocarbons have demonstrated excellent chemical and physical stability.^15^ This scalable synthetic methodology yields fluorine-free polymers with low-cost processing^16,17^ and a wide range of ion exchange capacities, making them highly suitable for membrane applications. Among these hydrocarbon-based membranes, isatin-derived polymers have been primarily investigated for gas separation,^18,19^ and proton exchange membranes (PEMs).^20–22^ More recently, their application in electrochemical devices has expanded to include both anion and cation exchange membranes.^23,24^

In this work, we expand upon these studies by synthesizing and characterizing polymers derived from biphenyl and isatin monomers, along with their sulfonated analogues, to further explore their application as cation exchange membranes for pH neutral RFBs. The incorporation of –SO_3_^−^ groups in the polymer backbone increases hydrophilic character of the polymer, facilitating ion transport in aqueous media by electrostatic interactions. To investigate the ionic transport properties, we prepared membranes with varying degrees of sulfonation and performed conductivity measurements for Li^+^, Na^+^, and K^+^ ions using chloride salt solutions, showing the highest conductivity for LiCl solution. Nevertheless, measuring the conductivity of a solution is a bulk technique and cannot directly quantify the transport of a specific ionic species across the membrane. Identifying which chemical species are charge carriers is essential for understanding and tuning the membrane's selectivity. To obtain such level of chemical specificity, techniques that can distinguish individual ionic species are required. Nuclear magnetic resonance (NMR) offers this capability. As an atom-specific and non-invasive technique, NMR can track selected nuclei *in situ*, providing direct insight into ion transport and speciation. For example, Latchem and colleagues recently demonstrated an inline ^1^H NMR method to characterize the crossover of redox-active species in an aqueous organic RFB.^25^ They demonstrated that the extent of crossover of redox-active species can depend strongly on the applied current, with migration contributing significantly under charging conditions, even at modest current densities.

Previously, we used ^7^Li NMR to identify and measure lithium-ion (Li^+^) migration through a dibenzodioxin-based microporous polymer, also evaluated as an RFB membrane alternative.^26^ However, these measurements were conducted without battery charging and discharging. To identify and quantify ion transport under battery operating conditions, we applied the synthesized biphenyl-isatin membranes in a symmetric iron-based aqueous redox flow system. In this configuration the Li^+^ quantification during cell cycling presents additional challenges: the continuous generation and consumption of the paramagnetic ferricyanide ions affect the spin relaxation of Li^+^ as a function of state-of-charge, and thus the ^7^Li NMR signal intensity and quantification.

Building upon these demonstrations of NMR's ability to elucidate crossover processes, we extended its application here to examine the transport of charge-balancing ions. Here, we address the challenge of relaxation attenuation using a quantitative relaxation correction. We first performed *operando*^7^Li NMR during battery cycling and applied Evans' method to estimate the ferricyanide ion concentration from the ^7^Li chemical shift. Next, we implemented a calibration procedure to correct for relaxation effects induced by ferricyanide ions. This enabled us to quantify the transport of Li^+^ ions through the membranes as a function of charge–discharge cycles. Using this protocol, we identified Li^+^ as the primary charge-balancing ion. The 65% sulfonated membrane exhibiting the highest performance was subsequently evaluated in a symmetric ferro/ferricyanide redox flow cell to assess its long-term cycling stability. The choice of a symmetric cell using a single redox-active couple is to avoid complication of electrolyte degradation. The results show a capacity retention of 92% over 180 cycles, demonstrating the promise of fluorine-free sulfonated polymers as viable and sustainable alternatives to perfluorinated membranes in electrochemical technologies.

## Materials and methods

### Chemicals

Biphenyl (BP, purity ≥99%), 2,3-dioxo-2,3-dihydroindole or isatin (IS, purity >97%), trifluoromethanesulfonic acid (TFSA, reagent grade, 98%), anhydrous potassium carbonate (K_2_CO_3_, purity = 99.995%), anhydrous chloroform (CHCl_3_), 1,3-propanesultone (PrS) and deuterated chloroform (CDCl_3_) were purchased from Sigma-Aldrich. BP and IS were stored in a desiccator at 35 °C under vacuum, while TFSA was stored in a refrigerator. K_2_CO_3_ was heated to 150 °C prior to use. Solvents such as dimethyl formamide (DMF) and dimethyl sulfoxide (DMSO), and salts, sodium hydride (NaH), lithium chloride (LiCl), potassium chloride (KCl), sodium chloride (NaCl) were used as received from Sigma-Aldrich. MilliQ water (18 MΩ cm) was used in all the salt solutions. Lithium ferrocyanide (Li_4_Fe(CN)_6_) and lithium ferricyanide (Li_3_Fe(CN)_6_) salts were prepared by ion exchange of the corresponding potassium salts respectively, which were purchased from Sigma-Aldrich. The cation exchange procedure was carried out by following a reported procedure,^27^ using a Dowex 50 cation-exchange resin (Sigma-Aldrich).

### Polymer synthesis, post-modification, and membrane preparation

BP (5 g, 32.4 mmol) and IS (5.48 g, 37.3 mmol) monomers, and anhydrous CHCl_3_ (28.2 mL) were charged into a three-neck, pear-shaped flask equipped with a mechanical stirrer and a nitrogen inlet and outlet. After the monomers dissolved, the solution was cooled with an ice bath. Then, TFSA (56.3 mL) was added dropwise using a dropping funnel. Subsequently, the ice bath was removed, and the temperature was raised to 25 °C using a controlled-temperature silicon bath. The reaction was allowed to proceed for 4 h until a highly viscous solution was obtained. The solution was poured into a methanol/deionized (DI) water mixture (2 : 1) under magnetic stirring. The resultant polymer fibers were sequentially washed with cold and hot DI water to remove the TFSA excess and finally washed with methanol. Then, the polymer was dried at 60 °C, dissolved in DMF, precipitated, washed with methanol and, finally, dried at 120 °C in a vacuum oven. The polymer is hereafter referred to as BPIS. *N*-Sulfopropylation of BPIS was conducted by reacting the secondary amides of the lactam rings with the PrS. The reaction conditions were optimized to achieve the desired sulfonation degree (SD). Two bases, NaH and K_2_CO_3_, were evaluated; however, no significant difference in SD was observed, and anhydrous K_2_CO_3_ was selected for subsequent reactions due to its ease of handling. In contrast, the molar ratio of reactants relative to the N–H groups per BPIS monomer unit (1 : PrS : Base) had a strong influence on SD. Ratios of 1 : 1.50 : 1.60, 1 : 2.43 : 1.60, and 1 : 3.03 : 1.60 yielded polymers with SDs ranging from 20% to 70%.

In a typical procedure, BPIS (0.3 g) was dissolved in anhydrous DMF (8 mL) in a 25 mL three-neck round-bottom flask equipped with mechanical stirring and maintained under a gentle nitrogen flow. The solution was cooled in an ice bath, and finely powdered anhydrous K_2_CO_3_ was added. After removing the ice bath, PrS was added, and the reaction mixture gradually heated to 60 °C and stirred for 48 h. The product was then poured onto a leveled glass plate placed on a hot plate at 80 °C to remove the solvent. The resulting solid was sequentially washed with hot deionized (DI) water, a DI water/acetone mixture (2 : 1 v/v), and acetone to remove residual K_2_CO_3_ and unreacted PrS. The sulfonated polymer was subsequently dissolved in DMSO, precipitated in acetone, and dried under vacuum at 80 °C for 12 h. The resulting polymers are referred to as BPIS–SD, where SD denotes the sulfonation degree.

Non-sulfonated and sulfonated films were prepared by casting from polymer solutions (200 mg of polymer in 8 mL DMSO). After dissolving the polymer, the solution was poured onto a glass ring placed on a heated, levelled glass plate at a temperature of 60 °C, until the solvent was evaporated. The resulting film was then peeled from the glass plate and subjected to a vacuum drying process at 80 °C for 10 hours. Films measuring 95 cm^2^ in area and 30 µm in thickness were obtained.

Further details related to the preparation and characterization of the polymers and membranes can be found in the SI. The membranes' mechanical strength, swelling ratio, morphology, and elemental analysis are included in Fig. S7–S12 and Tables S3–S5.

### Electrochemical impedance spectroscopy

To assess the conductivity of the membranes, Electrochemical Impedance Spectroscopy (EIS) was performed using a multichannel potentiostat/galvanostat (Biologic SP-300) at open potentials. A frequency ranged from 7 MHz to 10 mHz was investigated using an AC bias of 10 mV, with 20 data points acquired per decade. Membrane samples were soaked in 1 M LiCl solution for 24 hours prior to measurement after which they were then placed between two stainless steel electrodes in Swagelok cells. Impedance measurements were recorded in a temperature range from 25 to 80 °C controlled within FD023-230V BINDER oven.

Potentiostatic EIS (PEIS) was conducted to track changes in membrane resistance during galvanostatic cycling. Measurements were taken over a frequency range of 1 MHz to 100 mHz with 10 points per decade. An initial PEIS spectrum was recorded before cycling, and subsequent measurements were performed every twenty cycles to monitor the cell's evolution over long-term operation (more details can be found in Section S6 in the SI).

### Dialysis diffusion studies

Dialysis diffusion experiments using H-shaped cells at room temperature were conducted to assess the transport properties of BPIS membranes. The membrane was positioned between the tanks using phenolic screw caps and Teflon sealing rings. Each stirred tank was filled with a 15 mL solution. For LiCl, NaCl, KCl and Li_3_Fe(CN)_6_, the feed side was filled with 1 M aqueous solution, while the permeate side contained only DI water. A conductivity probe was inserted into the permeate compartment to measure the conductivity. The permeability assessment, along with the equations used, and the calibration procedure, are included in Fig. S14 and Table S7 in the SI.

### Battery cycling

Symmetrical flow battery charge–discharge cycling was conducted to assess the performance of the membrane and the transport of Li^+^. The Li_3_Fe(CN)_6_ and Li_4_Fe(CN)_6_ redox couple was selected due to its well-known stability, good solubility and fast redox kinetics in aqueous RFBs (ARFBs).^28^ External tanks were filled with 25 mL of 0.1 M redox active solution where Li_4_Fe(CN)_6_ served as the catholyte, and Li_3_Fe(CN)_6_ as the negolyte, respectively. The RFB cell was purchased from Jena Batteries GmbH (Germany). The electrodes were composed of two porous carbon felt electrodes (Sigracell GFA 6EA) of 5.30 cm^2^ in nominal area. The polymer membrane, which has an active area of 5.29 cm^2^, was placed between the electrodes. Galvanostatic charge and discharge tests with a potential range of −0.8V to 0.8V were conducted at three current densities (10 to 40 mA cm^−2^). Extended charge and discharge experiments over 180 cycles were conducted to evaluate membrane stability at neutral pH using an SP-300 Potentiostat (Biologic).

### Inline *operando*^7^Li NMR experiments

#### Acquisition parameters

Inline ^7^Li NMR spectra were acquired in H_2_O using a benchtop Bruker Fourier80 spectrometer (80 MHz, 1.88 T). The spectral acquisition was achieved by conducting single pulse-acquire experiments with a pulse angle of 90°. Data acquisition began after complete filling of the flow cell with electrolyte, which occurred approximately 50 s after initiating flow with the peristaltic pump. Prior to experiments, a 1 M LiCl solution with 100 mM of Li_3_Fe(CN)_6_ was used for calibration to determine relevant NMR parameters and ensure sufficient resolution. The spin-lattice relaxation time (*T*_1_) was experimentally determined to be 9 s by an inversion recovery pulse sequence. Ideally, this would require a recycle delay (d1) of at least 45 s to allow close-to-full relaxation between scans. However, because the experiment aimed to monitor electrolyte changes in real time during battery cycling, using a long recycle delay would have compromised the temporal resolution. To balance the need for time resolution with signal intensity, a d1 of 15 s and 8 scans (ns = 8) were used. The acquisition time (aq) was 2.5 s, resulting in one ^7^Li spectrum every 140 s ((d1 + aq) × ns = 140 s). While this approach leads to partial saturation and signal attenuation, the effects were quantified and corrected using a separate calibration, described more in detail in the following subsections and in Section S.4 in the SI.

#### Evans method

Based on Evans method,^29^ the change of chemical shift (Δ*δ*) of a spectator species is related proportionally to the concentration of a paramagnetic species (*C*_para_), following the simplified eqn (1):

1 Δ*δ* = constant·*C*_para_

This relationship enables experimental calibration using standard solutions of known paramagnetic species concentrations. To determine the constant, five solutions containing 1 M LiCl (as a constant source of ^7^Li nuclei) were prepared with an increasing concentration of K_3_Fe(CN)_6_ (0, 0.025, 0.050, 0.075 and 0.1 M). We chose to use the potassium ferric salt instead of the substituted, lithiated one, to have a known constant concentration of Li^+^ in solution. The solutions were measured with the same acquisition parameters as mentioned before, at a flow rate of 4 mL min^−1^. The chemical shift of ^7^Li was extracted directly from the processed spectra (Fig. 6A).

#### Relaxation correction method for Li^+^ quantification

As the electrolyte solution flows from the electrochemical cell into the detection region of the NMR instrument, its initial magnetization is essentially close-to-zero. Magnetic polarization occurs progressively as the solution moves through the magnetic field, but full magnetization (*i.e.*, thermal equilibrium) typically requires approximately five times of *T*_1_. At the chosen flow rate of 4 mL min^−1^, the residence time of the sample within the detection region is approximately 6 s. This is considerably shorter than the *T*_1_ value recorded for a 1 M LiCl solution with 100 mM of Li_3_Fe(CN)_6_, corresponding to 9 s. As a result, the flowing electrolyte does not have sufficient time to reach full magnetization before detection, leading to a systematic attenuation of the ^7^Li signal intensity. This effect became especially evident during battery operation. As the battery charged, the ^7^Li signal intensity was expected to increase, reflecting the accumulation of Li^+^ in the reduced electrolyte compartment. Instead, a decrease in intensity was observed caused by changes of *T*_1_ relaxation as the paramagnetic Fe^3+^ is being reduced to the diamagnetic Fe^2+^. To correct this magnetization attenuation, we developed a relaxation correction protocol to recover the actual Li^+^ signal intensity.

A series of ^7^Li NMR spectra were acquired from 1 M LiCl solutions containing varying concentrations of Fe^3+^, under identical flow and acquisition parameters to those used in the battery experiment. Since Fe^3+^ is paramagnetic and shortens *T*_1_, with this calibration we can effectively reproduce the range of paramagnetic relaxation present in the working battery. We define a relaxation correction parameter, *I*_corr_, as the ratio of the measured signal intensity at the presence of Fe^3+^ to the intensity without Fe^3+^. The resulting correction parameter exhibited an exponential dependence on Fe^3+^ concentration, in agreement with the exponential character of magnetization buildup. Because of this, the data were fitted to an exponential model, with a high degree of correlation (*R*^2^ = 0.99), as shown in Fig. 6B.

2 *I*_corr_ = 0.20 (5.79 − *e*^−[Fe^3+^]/34.43 mM^)

We extracted the Fe^3+^ concentration of our experiment using the fitting for the Evans method (SI Section S.4.2). Introducing these values in the fitted model returns the expected correction parameter (*I*_corr_). This allowed us to correct the measured intensity extracted from the processed spectra (*I*_apparent_) and obtain the true Li^+^ signal intensity (*I*_real_) by using the relationship in eqn (3):

3 *I*_real_ = *I*_apparent_·*I*_corr_^−1^

Once *I*_real_ is obtained, the Li^+^ concentration is determined using a linear calibration established under flow conditions (Fig. 6C). If our relaxation correction method is accurate, extracting the Li^+^ concentration from the integrated ^7^Li signal using a static (non-flow) calibration should yield same results to our proposed methodology. To validate the ^7^Li intensity correction method, we performed an inline experiment using aqueous solutions of 0.1 M Li_4_Fe(CN)_6_ and 0.1 M Li_3_Fe(CN)_6_ with 1 M LiCl as the supporting electrolyte. In this experiment, the battery was charged at a current density of 15 mA cm^−2^ and a flow rate of 4 mL min^−1^. Following the charge step, an additional rest period with no current and no flow was introduced while NMR acquisition continued. The acquisition parameters and related information of the method validation are provided in the SI Section S.4.4, including the outcomes of said experiment (Table S8 and Fig. S17), detailed calculation steps, and the calibration used for the static measurement (Fig. S18). The flow and static measurements indeed yield the same Li^+^ concentration.

## Results and discussion

### Synthesis and characterization of the BPIS membrane

**Fig. 1:**
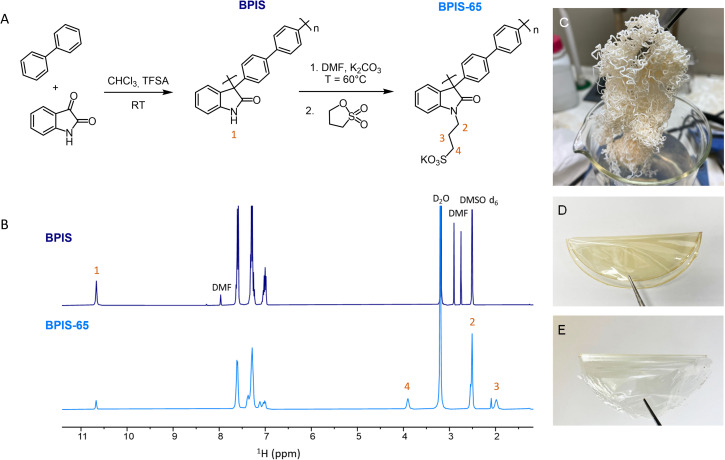
(A) Schematic representation of the synthetic route for BPIS and sulfonated BPIS–SD polymer derivatives. (B) Polymer fibers of BPIS. (C) ^1^H-NMR spectra of BPIS (top) and BPIS–65 (bottom) at 400 MHz in DMSO-d_6_. (D) Self-standing BPIS membrane casted in DMF. (E) Self-standing BPIS–65 membrane casted in DMSO.

**Fig. 2:**
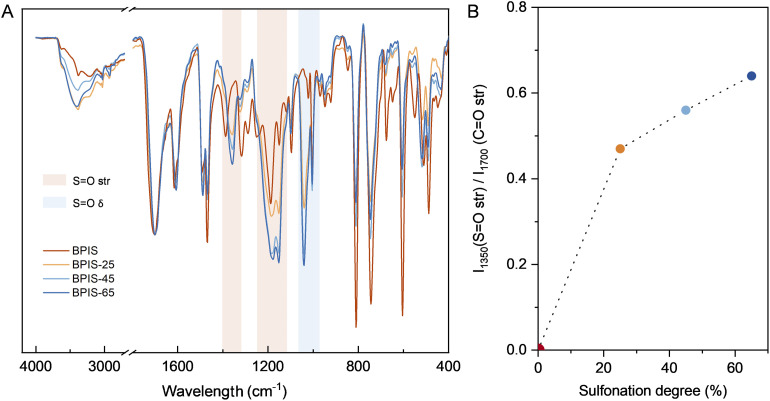
(A) FTIR spectra of BPIS polymers films. All sulfonated polymers showed characteristic absorption bands of sulfonate groups at 1350, 1175 and 1150 cm^−1^ (S=O highlighted in pink) and 1040 cm^−1^ (S–O highlighted in blue), and an additional band at 1525 cm^−1^ (CH_2_*δ*) due to the methylene's included in the sulfonated chain. (B) Intensity of the band at 1350 cm^−1^ as a function of the sulfonation degree. All the spectra were normalized to 1700 cm^−1^ band (C=O). Increasing intensity of 1175 and 1150 cm^−1^ band (S=O) is caused by the sulfonation of the polymer.

As illustrated in Fig. 1A, the BPIS polymers were synthesized through a superacid-catalyzed Friedel–Crafts polycondensation, followed by a post-synthetic modification to introduce ion exchange groups.^15,18,21,30,31^ The superacid-catalyzed polyhydroxylakylation reactions have been conducted under stoichiometric and non-stoichiometric conditions, following literature procedures.^18,21,32^ Notably, prior studies have shown that non-stoichiometric conditions can accelerate the reaction.^33^ The polymerization reaction of BPIS was conducted using an BP : IS molar ratio of 1 : 1.15 at room temperature for 4 h, resulting in polymer (Fig. 1B) with an inherent viscosity of 1.76 ± 0.01 dL g^−1^. The sulfonation of BPIS polymers was successfully achieved obtaining polymers with varying sulfonate content. A comparison of the ^1^H-NMR spectra for BPIS and BPIS–65 (post-sulfonation, SD = 65%) is shown in Fig. 1C. The SD of BPIS-SD was quantified by comparing the integrated areas of the aromatic proton peaks (–C_ar_–H: 6.88–7.70 ppm) or the methylene proton peak (–CH_2_: 3.90 ppm) to that of the secondary amide proton peak ( <svg xmlns="http://www.w3.org/2000/svg" version="1.0" width="10.400000pt" height="16.000000pt" viewBox="0 0 10.400000 16.000000" preserveAspectRatio="xMidYMid meet"><metadata> Created by potrace 1.16, written by Peter Selinger 2001-2019 </metadata><g transform="translate(1.000000,15.000000) scale(0.011667,-0.011667)" fill="currentColor" stroke="none"><path d="M80 1160 l0 -40 40 0 40 0 0 -40 0 -40 40 0 40 0 0 -40 0 -40 40 0 40 0 0 -40 0 -40 40 0 40 0 0 -40 0 -40 40 0 40 0 0 -40 0 -40 40 0 40 0 0 -40 0 -40 40 0 40 0 0 80 0 80 -40 0 -40 0 0 40 0 40 -40 0 -40 0 0 40 0 40 -40 0 -40 0 0 40 0 40 -40 0 -40 0 0 40 0 40 -40 0 -40 0 0 40 0 40 -80 0 -80 0 0 -40z M560 520 l0 -40 -40 0 -40 0 0 -40 0 -40 -40 0 -40 0 0 -40 0 -40 -40 0 -40 0 0 -40 0 -40 -40 0 -40 0 0 -40 0 -40 -40 0 -40 0 0 -40 0 -40 -40 0 -40 0 0 -40 0 -40 80 0 80 0 0 40 0 40 40 0 40 0 0 40 0 40 40 0 40 0 0 40 0 40 40 0 40 0 0 40 0 40 40 0 40 0 0 40 0 40 40 0 40 0 0 80 0 80 -40 0 -40 0 0 -40z"/></g></svg> N–H: 10.67 ppm), which served as an internal reference that was normalized to one proton. Variations of up to 10% were observed between the area ratios obtained from C_ar_–H signals or –CH_2_ signal. The SD of the BPIS–SD polymers was found to range between 20 and 70%. NMR spectra of all synthesized polymers can be found in Fig. S1–S4. The inherent viscosity for BPIS–65 was 3.87 ± 0.01 dL g^−1^, which was superior to that BPIS (Table S1). Non-sulfonated and sulfonated polymers formed self-standing membranes by casting (Fig. 1D and E) with good mechanical properties (Table S3, and Fig. S7), facilitating easy integration in the electrochemical flow cells. The membranes were characterized using Attenuated Total Reflectance Fourier Transform Infrared (ATR-FTIR) spectroscopy (Fig. 2A). The BPIS spectrum exhibited the characteristic absorption bands of lactam rings at 3382 cm^−1^ (N–H), 3020 cm^−1^ (C–H, aromatic-H), 1700 cm^−1^ (C=O), and 1600 cm^−1^ (N–H *δ*). Aromatic carbons (C_ar_–C_ar_) vibrations were assigned to 1617, 1495, 1470 cm^−1^. The presence of additional bands appearing at 1525 cm^−1^ (CH_2_*δ*), 1350, 1175 and 1150 cm^−1^ (S=O), and 1040 cm^−1^ (S–O) in the BPIS–SD spectra confirmed the *N*-sulfopropylation of the BPIS. To give an illustrative example, Fig. 2B shows the intensity of the band associated with sulfonate groups at 1350 cm^−1^ as a function of sulfonation. The thermal stability of membranes was determined by Thermogravimetric Analysis (TGA), showing high degradation onset temperatures (>450 °C). Further details about measurements and the results are provided in the SI (Fig. S5, S6 and Table S2), as well as the assessment of water uptake and swelling ratio (Fig. S8 and Table S4). The morphology of the various membranes was examined using SEM and EDX characterization, as presented in Fig. S9–S12.

### Membrane conductivity measured by EIS

**Fig. 3:**
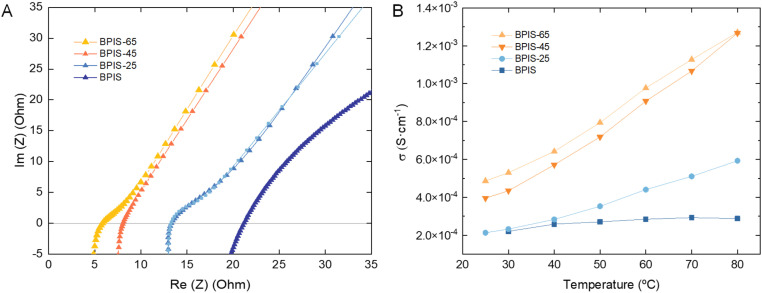
(A) Nyquist plot for polymeric films studied at 25 °C. (B) Conductivity of BPIS–SD membranes in LiCl solutions, as functions of operating temperature. Membrane resistance was determined from the intersection on the real axis in the high-frequency region.

The membrane conductivity was calculated from the resistance values obtained at various temperatures by applying the following eqn (4):

4 *σ* = *e*·A^−1^·*R*_m_^−1^

where *σ* is the ionic conductivity of the membrane in S cm^−1^, *e* the thickness of each sample in cm, *A* is the effective membrane area of 1.13 cm^2^, and *R*_m_ the membrane resistance in Ω obtained from intersection with the real axis in the Nyquist plots (Fig. S13), based on the fitting of the data with the equivalent electric circuit L1 + R1/C1 + Q1. The data analysis is shown in Table S6 in the SI. As observed in Fig. 3A, the membrane resistance decreases significantly with increasing SD. The non-sulfonated BPIS membrane demonstrates greater resistance compared to other samples, whereas BPIS–65, which has the highest SD, displays the lowest measured resistance. This trend aligns with the conductivity data in Fig. 3B, where samples underwent a temperature ramp. This suggests that ion transport is strongly influenced by the presence of –SO_3_^−^ groups within the membrane. It is well established that ion transport through cation-exchange membranes is governed by mechanisms such as structural diffusion and Donnan exclusion.^34^ Our observations indicate that these anionic sites facilitate mass transport through electrostatic interactions, thereby improving overall ion mobility and reducing the overall resistance of the membrane.

### Ion permeability *via* conductivity and NMR measurements

**Fig. 4:**
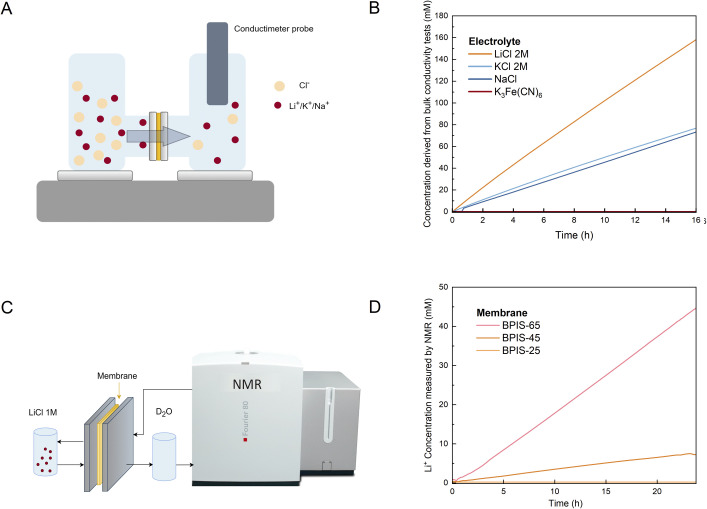
(A) Schematic illustration for small ions through isatin derived polymeric membranes in a H-shaped cell configuration (left). (B) Diffusion of 1 M LiCl, NaCl, KCl, or K_3_Fe(CN)_6_ through BPIS–65 membrane measured in two compartment diffusion cells (right). (C) Scheme of the inline ^7^Li-NMR permeability set-up. The permeate tank only contained D_2_O and was connected directly to the instrument. (D) Li^+^ concentration in the permeate tank monitored for 24 h. Maximum Li^+^ concentration reached after 24 h was, respectively: 45 mM (BPIS–65), 6.5 mM (BPIS–45), and 0 mM (BPIS–25). The concentration curve of BPIS–25 is not visible as it nearly coincides with the *x*-axis.

The permeability of various supporting salts and the crossover of redox-active electrolytes were evaluated using conductivity measurement, and the setup is schematically illustrated in Fig. 4A. Common aqueous salts used in redox flow batteries were tested to assess ionic transport through the membrane BPIS–65, alongside an aqueous solution of K_3_Fe(CN)_6_, selected to evaluate the membrane's selectivity against larger redox-active species. The concentration of K_3_Fe(CN)_6_ in the permeate compartment remained constant over time (Fig. 4B), showing negligible crossover and proving the membrane's high selectivity for small ions. Notably, these values compared to the benchmark Nafion membrane are more than an order of magnitude lower, demonstrating limited crossover of these larger anions across the BPIS-65 membrane (SI Table S7). Permeability values of all the membranes assessed can be found in the SI Section S.3 (see Fig. S15). To identify the specific ions that pass through the BPIS membranes, we employed benchtop ^7^Li NMR to quantitatively monitor real-time changes in Li^+^ concentration under flow conditions. Inline experiments were conducted using a Bruker Fourier80 benchtop NMR spectrometer (80 MHz). The setup included a RFB cell positioned outside the magnet, equipped with carbon felt electrodes and the BPIS-SD membrane under study (Fig. 4C). Two external tanks were connected to the system following a previously reported procedure,^26^ with the feed tank containing a 1 M LiCl solution, while the permeate tank was filled with Milli-Q water. Driven by the concentration gradient, Li^+^ ions diffused across the membrane and were continuously circulated through the NMR flow apparatus. The resulting ^7^Li signal was acquired over a 24-hour period and quantified using a calibration procedure. The NMR results corroborated the diffusion measurements, showing that the degree of sulfonation in the polymer had a strong influence on ion transport. As illustrated in Fig. 4D, sulfonated membranes with higher sulfonate group content (BPIS–45 and BPIS–65) allowed measurable amounts of Li^+^ to cross the membrane, with BPIS–65 yielding the highest concentration. In contrast, the less modified BPIS–25 membrane showed no detectable Li^+^ signal even after 24 hours. The measured Li^+^ permeability *via* NMR and total permeability *via* conductivity are summarized in Table 1. The calibration for the flow NMR quantification is shown in Fig. 6C. BPIS–25 exhibited a low Li^+^ permeability (3.5 × 10^−9^ cm^2^ s^−1^), whereas BPIS–65 demonstrated a value of an order of magnitude higher (8.7 × 10^−8^ cm^2^ s^−1^). This enhancement is attributed to the higher density of negatively charged sulfonate groups (–SO_3_^−^), resulting from the dissociation of –SO_3_K under neutral pH. These fixed negative charges enhance cation transport *via* electrostatic interactions with species such as Li^+^.

**Table 1:** Permeability values for the different sulfonated BPIS membranes. Here we compare the permeability results from the bulk conductivity (*P*_total_) and those obtained from inline ^7^Li NMR (*P*_Li^+^_) measurements

Membrane	BPIS–25	BPIS–45	BPIS–65
P_total_ (cm^2^ s^−1^)	3.48 × 10^−9^	1.20 × 10^−8^	8.69 × 10^−8^
P_Li^+^_ (cm^2^ s^−1^)	1.56 × 10^−12^	2.33 × 10^−9^	1.40 × 10^−8^

Consequently, higher sulfonation levels directly correlate with improved ion permeability.

However, in aqueous environments, the transport of both cations and anions is necessary to maintain electroneutrality across the membrane. Especially at high salt concentrations, such as the 1 M LiCl used in this study, the formation of contact ion pairs and/or solvated aggregates may also occur. These species can facilitate the co-transport of ions and may contribute to the elevated overall permeability values.^26,37^ The difference between the total ion and the Li^+^ cation permeability may thus be rationalized by presence of a transport *via* ion pairs (Table 1).^38^ Another possibility is that H^+^ cations diffuse in the opposite direction of Li^+^ to maintain the charge neutrality, resulting in an increased concentration of OH^−^and thus an increase in the total conductivity of the measured compartment. In addition, the different cell designs, and flow conditions applied in these measurements might introduce additional variations.

### *Operando* NMR measurements of state-of-charge and charge-balancing ions

**Fig. 5:**
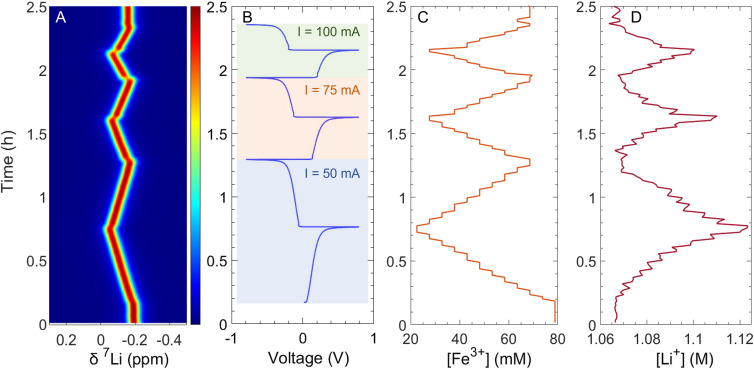
(A) Inline *operando* pseudo-2D ^7^Li NMR spectra, (B) voltage profile recorded at three different current densities (10, 15, and 20 mA cm^−2^) for an electrode with a nominal surface area of 5 cm^2^, (C) Fe^3+^ concentration, and (D) Li^+^ concentration in the electrolyte, extracted from the observed ^7^Li NMR spectra using chemical shift analysis and a calibration procedure, respectively. Results obtained during battery cycling of a full-symmetric flow cell containing an electrolyte with 80 mM Li_3_Fe(CN)_6_ against 80 mM Li_4_Fe(CN)_6_.

**Fig. 6:**
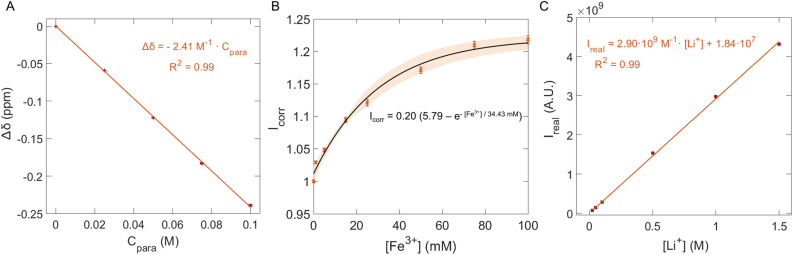
(A) Linear fitting between the Fe^3+^ concentration (*C*_para_) and the observed chemical shift difference (Δ*δ*). The strong linear correlation confirms the suitability of the Evans method under our experimental conditions. (B) Correction parameter, *I*_corr_, derived from a series of calibrating solutions with various Fe^3+^ concentrations. As the concentration of Fe^3+^ increased, each individual ^7^Li spin had shorter *T*_1_, accumulating more magnetization, leading to a significant increase in the NMR signal intensity. The correction parameters were defined as the ratio of the measured signal intensity with Fe^3+^ to that without Fe^3+^ under flow. The shaded region represents the combined deviation between each experimental data point and the fit. (C) Linear fitting between the integrated signal intensity, *I*_real_, of the calibration solutions with various LiCl concentrations at a flow rate of 4 mL min^−1^.

**Fig. 7:**
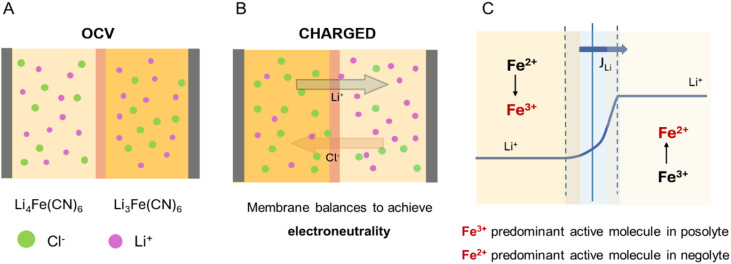
Schematic illustration of ion distribution from the supporting electrolyte under different conditions: (A) Open Circuit Potential (OCV), (B) during the charging process of the battery. (C) Li^+^ concentration profile throughout the charging period. The predominant redox-active species in each electrolyte is highlighted in red, whereas *J*_Li_ is the diffusive flux of Li^+^ cations.

After evaluating each membrane's Li^+^ permeability, the membrane exhibiting the best transport properties (BPIS–65) was studied in a symmetric flow cell connected to our inline benchtop NMR set-up. *Operando* experiments were performed using aqueous solutions of 0.08 M Li_4_Fe(CN)_6_ and Li_3_Fe(CN)_6_ with 1 M LiCl as a supporting electrolyte. The Li_3_Fe(CN)_6_ solution circulated through the NMR system to monitor real-time changes in Li^+^ concentration. The electrochemical protocol involved three galvanostatic charge–discharge cycles at three constant current values. Fig. 5 presents time-synchronized battery voltage profile and NMR spectra, revealing a strong correlation between them. Initially, the battery was left to rest to observe the chemical shift of Li^+^ in the absence of applied current (Fig. 5A). Once charging began at around 8 minutes (Fig. 5B), the ^7^Li signal shifted towards higher chemical shift, indicating the start of the reduction of [Fe(CN)_6_]^3−^. The paramagnetic nature of [Fe(CN)_6_]^3−^ ions (Fe^3+^), in contrast with the diamagnetic [Fe(CN)_6_]^4−^ counterpart (Fe^2+^), led to changes in the bulk magnetization of the electrolyte solution during charge and discharge. As the relative concentrations of these species change, the bulk magnetization changes, affecting the ^7^Li resonant frequency of Li^+^ cations in solution. Over time, this shift can be used to determine the concentration of paramagnetic ions in solution using the well-established Evans method.^29^ The linear fit of paramagnetic concentration and chemical shift is shown in Fig. 6A. Here we applied Evans method to extract the concentration of Fe^3+^ in solution throughout battery operation,^35,36^ thus measuring the state of charge of the battery (Fig. 5C). In addition to influencing chemical shifts, paramagnetic species significantly affect nuclear spin relaxation, enhancing both longitudinal and transverse relaxation rates, resulting in shorter relaxation times. In this system, the changing concentration of Fe^3+^ ions alters relaxation dynamics as the experiment progresses, affecting the measured ^7^Li signal intensity and complicating its direct quantification *via* signal integration. To account for this relaxation effect, a protocol was developed to correct for incomplete magnetization build-up (Fig. 6B). The apparent signal intensity (*I*_apparent_) measured under flow is corrected using eqn (3), yielding the real signal intensity (*I*_real_). This corrected intensity is then converted to a Li^+^ concentration using the calibration shown in Fig. 6C. When [Fe(CN)_6_]^3−^ is reduced to [Fe(CN)_6_]^4−^, an equivalent positive charge must be balanced across the membrane, normally by migration of a charge-balancing cation from the opposite half-cell (Fig. 7). We then observe that Li^+^ ions migrate toward the tank with a higher concentration of Fe^2+^ to compensate for the deficiency of positive charges. Table 2 summarizes the comparison between the amount of Fe^3+^ consumed and the corresponding concentration of Li^+^ detected at the end of three charge cycles. At 10 mA cm^−2^ and 15 mA cm^−2^, the amount of consumed Fe^3+^ is equal to the amount of migrated Li^+^ cations, showing that Li^+^ is the principal charge-balancing ion. Interestingly, a difference is observed for the last cycle at the highest current (20 mA cm^−2^), suggesting that Li^+^ alone does not fully compensate the charge transport under these conditions. A slight broadening of the ^7^Li resonance was detected at this increased charging rate (Fig. S16), especially during the transition when the Fe^3+^ concentration shifts quickly. This dynamic broadening arises from chemical shift changes occurring on a timescale comparable to the NMR acquisition. Although this effect may slightly decrease the observed Li^+^ signal, its influence remains minor since the concentration was determined by integrating over the entire resonance.

**Table 2:** Concentrations of consumed Fe^3+^ and corresponding Li^+^ detected at the end of each charging cycle. Relative uncertainties in Fe^3+^ detection were estimated from the FWHM of the ^7^Li NMR peak. The propagated uncertainty in the Li^+^ concentration from calibration was found to be negligible (<0.01 mM). The details of these uncertainty estimates are provided in Section S.4 of the SI

Charge current density	Consumed [Fe^3+^]	Balancing [Li^+^]
10 mA cm^−2^	56.35 ± 0.02 mM	57.04 mM
15 mA cm^−2^	40.98 ± 0.01 mM	41.36 mM
20 mA cm^−2^	40.98 ± 0.01 mM	32.69 mM

We therefore attribute the remaining discrepancy primarily to increased concentration polarization at the membrane interface. At elevated currents, ion accumulation near the membrane can lead to the formation of a boundary layer with a steep concentration gradient.^39^ This polarization limits Li^+^ transport across the membrane, and to maintain charge neutrality, other cations present in the electrolyte may migrate through the membrane to compensate for the restricted Li^+^ flux. While we cannot directly identify the compensating species, we speculate that protons from the solvent are the most plausible charge-carriers, warranting future investigation. The resulting imbalance could lead to increased cell polarization and reduced performance. In summary, *operando* NMR measurements revealed that Li^+^ cations were the predominant charge-balancing ions during battery cycling, although contributions from other cations seem to be significant at higher current densities.

### Battery cycling

**Fig. 8:**
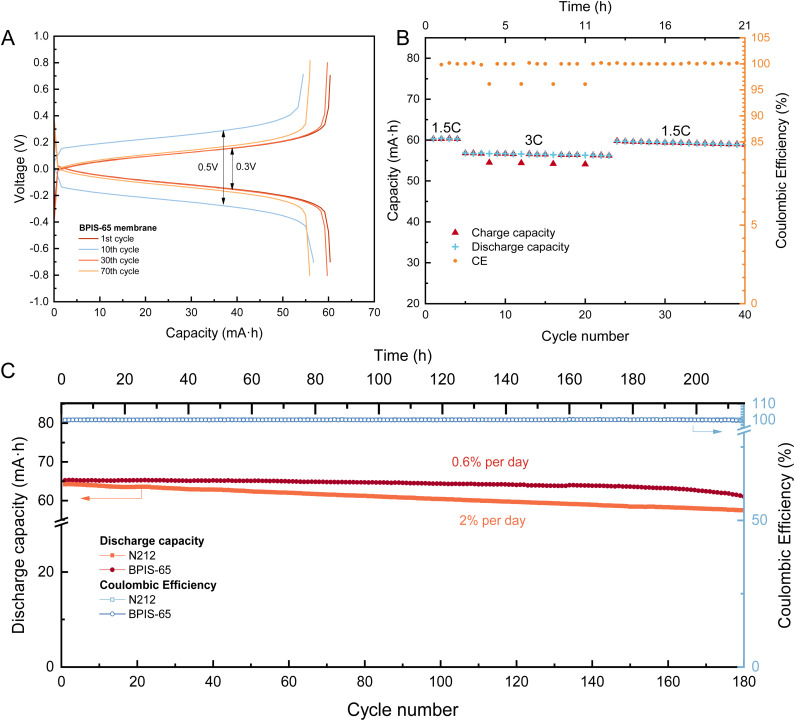
Performance metrics of an aqueous symmetric RFB consisting of 0.1 M of Li_4_Fe(CN)_6_ against 0.1 M of Li_3_Fe(CN)_6_. (A) Voltage profile obtained during cycling with a BPIS–65 membrane at different current densities: 20 mA cm^−2^ (for the 1–5th and 22–70th cycles), and 40 mA cm^−2^ (for the 5–22nd cycles). (B) Rate capability of BPIS–65 membrane at different current densities. (C) Comparison of long cycling stability and coulombic efficiency for two batteries containing different membranes: BPIS–65 and N212. Discharge capacity data were linearly fitted to derive capacity decay rates.

After identifying the charge-balancing ions through the BPIS–65 membrane, we assessed the stability of the membrane in another redox flow cell. Galvanostatic cycling was performed using 25 mL solutions of 0.1 M Li_4_Fe(CN)_6_ and 0.1 M Li_3_Fe(CN)_6_ respectively, which contained 1 M LiCl to be used as the supporting salt. The electrolytes were introduced into the electrochemical cell at a continuous flow rate of 50 mL min^−1^. Additionally, we also performed inline measurements to observe the conductivity changes during cycling (Fig. S19). The membrane exhibited good stability and capacity retention (Fig. 8A) during repeated cycles at various charging rates (Fig. 8B), demonstrating its robust performance under different operational conditions. When cycling at a higher current density of 40 mA cm^−2^ (cycles 5 to 22), the capacity utilization decreased slightly due to mass transport limitations (Fig. 8B), resulting in an increase in voltage polarization. However, when returning to a lower current of 20 mA cm^−2^, the capacity reverted to its prior levels, indicating that the membrane consistently exhibits stable electrochemical performance. Symmetric flow cells were continuously charged and discharged at a constant current density of 20 mA cm^−2^, achieving a capacity of 65 mA h (compared to the theoretical 67 mA h), completing a total of 180 cycles (Fig. 8C). This is further supported by the high coulombic efficiency values close to 100%. We observed a steady capacity fade overtime (Fig. S20), even though our neutral-pH and symmetric cell should in principle be largely immune to crossover or chemical degradation.^40^ Instead, we attribute this loss to progressive fouling of the BPIS–65 membrane,^41^ meaning the membrane's resistance increased over repeated cycling under galvanostatic control. The rising resistance elevates the overpotential at a fixed current, causing the cell voltage to hit its cut-off limits sooner on each charge and discharge process (Fig. S21A). Electrochemical Impedance Spectroscopy measurements were performed along galvanostatic cycling, revealing an increase in resistance as cycling progresses (Fig. S21B and S22). As a result, even though the active species concentration remains unchanged, the growing polarization across the membrane gradually cuts short the accessible voltage window, resulting in less capacity with each successive cycle.

## Conclusions

We demonstrated the potential of biphenyl-isatin-based polymers as cation exchange membranes for iron-based RFBs. A key finding is that increasing the sulfonated group content above 50% enhances selectivity and ionic permeability. In particular, the membrane containing 65% –SO_3_^−^ groups exhibited the highest selectivity for small charge-balancing cations like Li^+^ while effectively suppressing the crossover of larger redox-active species like K_3_Fe(CN)_6_. The BPIS–65 membrane was evaluated in a symmetric flow-cell configuration, showing good capacity retention and cycling stability under long-term galvanostatic operation. These results highlight the promise of all-sulfonated non-fluorinated polymer membranes as sustainable alternatives to per-fluorinated materials like Nafion. With further optimization, these materials could enable environmentally friendly, high-performance ion-selective membranes not only for aqueous RFBs but also for a broader range of electrochemical energy systems.

In parallel, we developed a benchtop *operando* NMR method to identify and quantify charge-balancing ions in a working symmetric flow cell. To mitigate the effects of paramagnetic species on relaxation times and signal intensity, we combined the Evans method with a relaxation correction protocol. This approach enabled accurate real-time tracking of Li^+^ ions, even in the presence of paramagnetic components such as ferricyanide. At low current densities, Li^+^ were the primary charge balancing cations, whereas at higher currents, a considerable deviation between charge and Li^+^ concentration was observed.

Overall, this work establishes benchtop NMR as a practical and powerful tool for *operando* studies of RFB systems, capable of resolving state-of-charge and ion transport phenomena *in situ* and simultaneously. Its low cost and ease of implementation make *operando* NMR broadly accessible to a wider research community and adaptable to commercial-scale systems. Moreover, the method is particularly suited to chemistries involving paramagnetic electrolytes, offering valuable insights for the rational design and optimization of next-generation ion-selective membranes.

## Author contributions

EWZ and JCG conceived the scientific idea. CA, AEL and MSR performed the polymer synthesis, membranes preparation, and membrane characterization with assistance from JCML and JCG. GST and MSR performed electrochemical characterization and *in situ* NMR experiments with the support of EWZ and JCG. GST and EWZ conducted the NMR analysis and developed the corresponding methodology. GST and MSR wrote, reviewed, and edited the original draft of the manuscript. EWZ, JCG, and CA supervised the work and contributed to the validation of the results. EWZ, JCG, CA and AEL provided funding acquisition. All authors have given approval to the definitive version of the manuscript.

## Conflicts of interest

The authors declare no competing financial interest.

## Supplementary Material

TA-014-D5TA06160A-s001

## Data Availability

The data supporting this article have been included as part of the supplementary information (SI). Supplementary information: membrane characterizations, permeability assessments, details of the *operando* NMR and conductivity experiments, the validation of the NMR relaxation correction methodology, and battery cycling data. See DOI: https://doi.org/10.1039/d5ta06160a.
