# Supplementary material for: Operando benchtop NMR study of ion transport through fluorine-free polymer membranes in a symmetric redox flow cell
Source: J Mater Chem A Mater. 2026 Feb 2;14(18):10800–12. doi: 10.1039/d5ta06160a (PMC12888836; doi:10.1039/d5ta06160a)
Supplement: TA-014-D5TA06160A-s001 [file TA-014-D5TA06160A-s001.pdf]

Supplementary Information (SI) for Journal of Materials Chemistry A.
This journal is © The Royal Society of Chemistry 2026

# Supporting Information

## Operando Benchtop NMR Study of Ion Transport through Fluorine-Free Polymer Membranes in a Symmetric Redox Flow Cell

Giu A. Silva Testa[‡a], Marta Santos Rodríguez[‡b], Juan Carlos Martínez-López[b], Ángel E. Lozano[b], Cristina Álvarez*[b], Javier Carretero-González*[b], Evan Wenbo Zhao*[a]

[a] Magnetic Resonance Research Center, Institute for Molecules and Materials, Radboud University, 6525 AJ Nijmegen, The Netherlands

[b] Institute of Polymer Science and Technology, ICTP, CSIC, C/Juan de la Cierva, 3, Madrid 28006, Spain

[‡] Equal contribution

*Correspondence email: evanwenbo.zhao@ru.nl; jcarretero@ictp.csic.es; cristina.alvarez@ictp.csic.es

# Contents

S1. Characterization Techniques: ........................................................ 3
S1.1. Sulfonation degree comparison between polymers via $^{1}H$-NMR ........................................................ 4
S1.2 Inherent viscosity ........................................................ 6
S1.3. Thermogravimetric analysis (TGA) of BPIS membranes ........................................................ 6
S.1.4. Tensile strength ........................................................ 8
S.1.5. Water Uptake and Swelling Ratio ........................................................ 8
S.1.6. Surface Morphology and Elemental Analysis ........................................................ 10
S.2 Electrochemical Impedance Spectroscopy (EIS) ........................................................ 13
S.3 Membrane permeability assessment ........................................................ 14
S.4 In-line operando $^{7}Li$ NMR experiments ........................................................ 17
S.4.1 Calibration for $Li^{+}$ quantification in flow ........................................................ 17
S.4.2 Evans method ........................................................ 17
S.4.3 Associated uncertainty in $Li^{+}$ quantification method ........................................................ 19
S.4.4 Validation of $Li^{+}$ quantification methodology ........................................................ 19
S.5 In-line conductivity measurement of the BPIS-65 half cell ........................................................ 22
S.6 Cycling performance of BPIS-65 symmetric cell ........................................................ 22

## S1. Characterization Techniques:

### Nuclear Magnetic Resonance

$^{1}H$-NMR spectra of polymers were recorded on a Bruker Avance spectrometer at a resonance frequency of 400 MHz at 60 °C and using a relaxation delay time of 10 s. Polymer samples (10-20 mg) were dissolved in 0.7 mL of deuterated dimethyl sulfoxide (DMSO $d_6$). DMSO-$d_6$ signal ($^{1}H$ 2.5 ppm) was used as internal reference.

### Infrared Spectroscopy

Membranes prepared by casting were characterized by Attenuated Total Reflectance-Fourier Transform Infrared (ATR-FTIR) spectroscopy on a PerkinElmer RX-1 FTIR instrument equipped with an ATR module. Measurements were taken over a wavenumber range of 400 to 4000 $cm^{-1}$, and each polymer spectrum was averaged from 64 scans per second.

### Thermogravimetric Analysis

Thermogravimetric analysis (TGA) of the membranes was conducted with a TA-Q500 thermobalance under a nitrogen flow of 60 $mL\cdot min^{-1}$. Thermogravimetric analysis was conducted at 20 $°C\cdot min^{-1}$ using a High-resolution (Hi-Res) mode in a temperature range from 30 °C to 850 °C. The sensitivity and resolution parameters of Hi-Res mode were set to 1 and 4, respectively.

### Tensile Strength

Mechanical properties of membranes were evaluated under uniaxial tensile tests at RT using an MTS Synergie-200 testing machine provided with a 100 N load cell. Rectangular test pieces of 5mm in width and 30mm in length were subjected to a tensile load applied at 5 $mm\cdot min^{-1}$ until fracture.

### S1.1. Sulfonation degree comparison between polymers via $^1$H-NMR

Figures S1-S4 show the NMR spectra of the non-sulfonated and sulfonated polymers synthesized in this work.

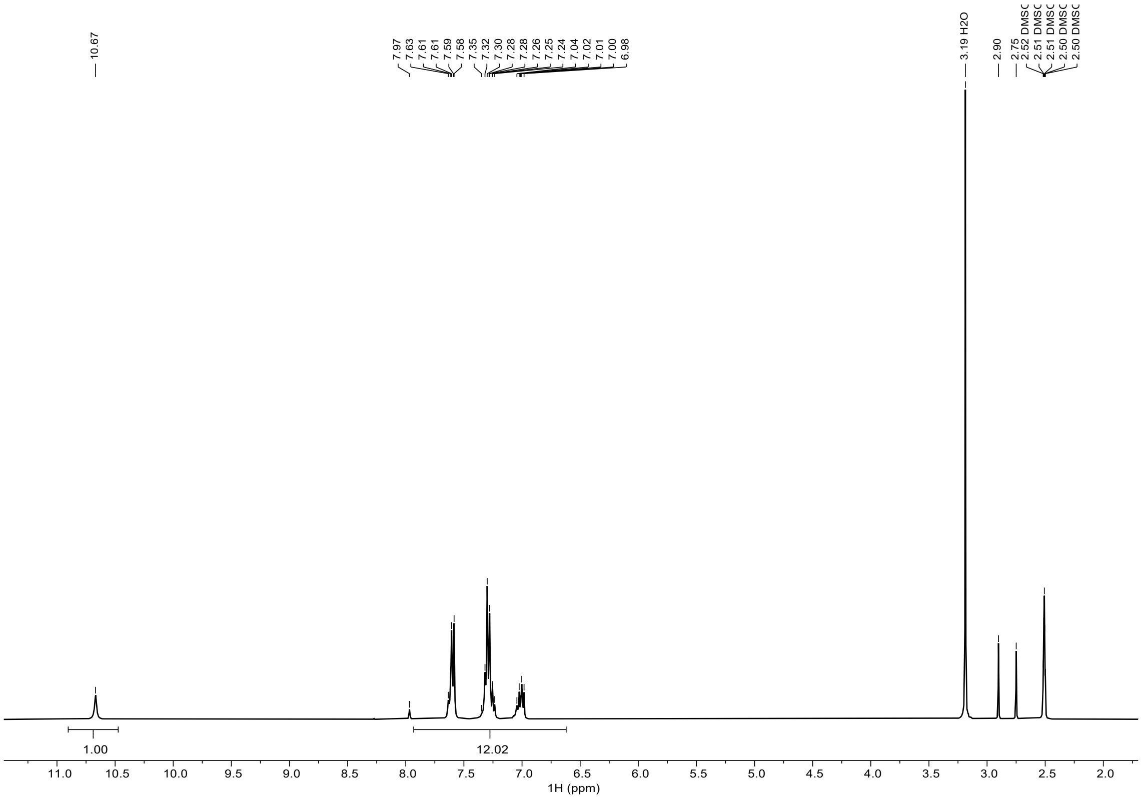


**Figure S1.** $^1$H-NMR (400 MHz, DMSO-d$_6$) spectrum of BPIS.

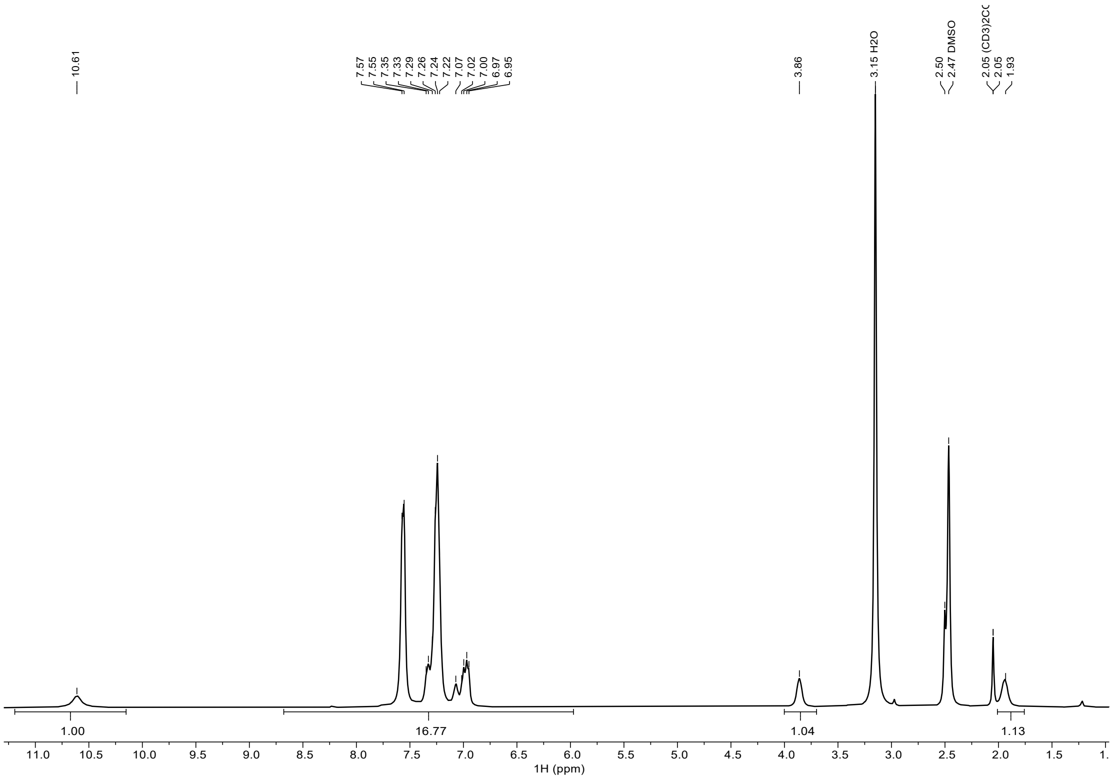


**Figure S2.** $^1$H-NMR (400 MHz, DMSO-d$_6$) spectrum of BPIS-25.

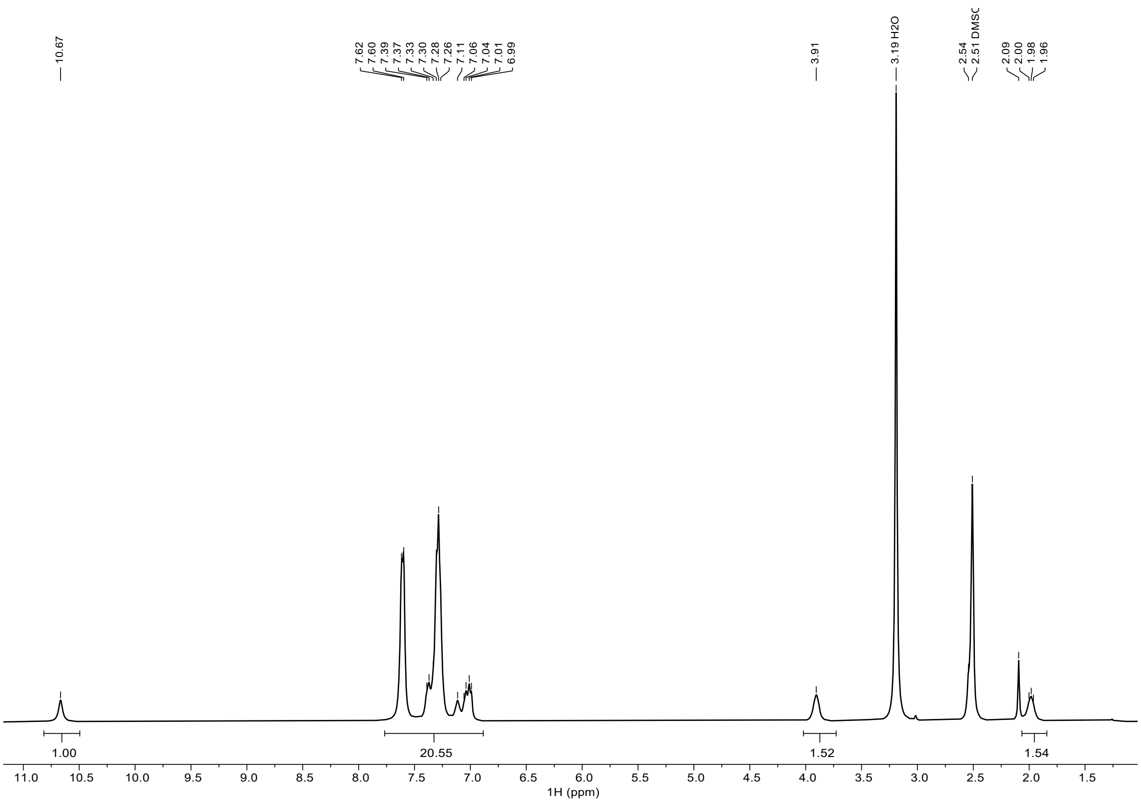


**Figure S3.** $^{1}H$-NMR (400 MHz, DMSO-$d_6$) spectrum of BPIS-45.

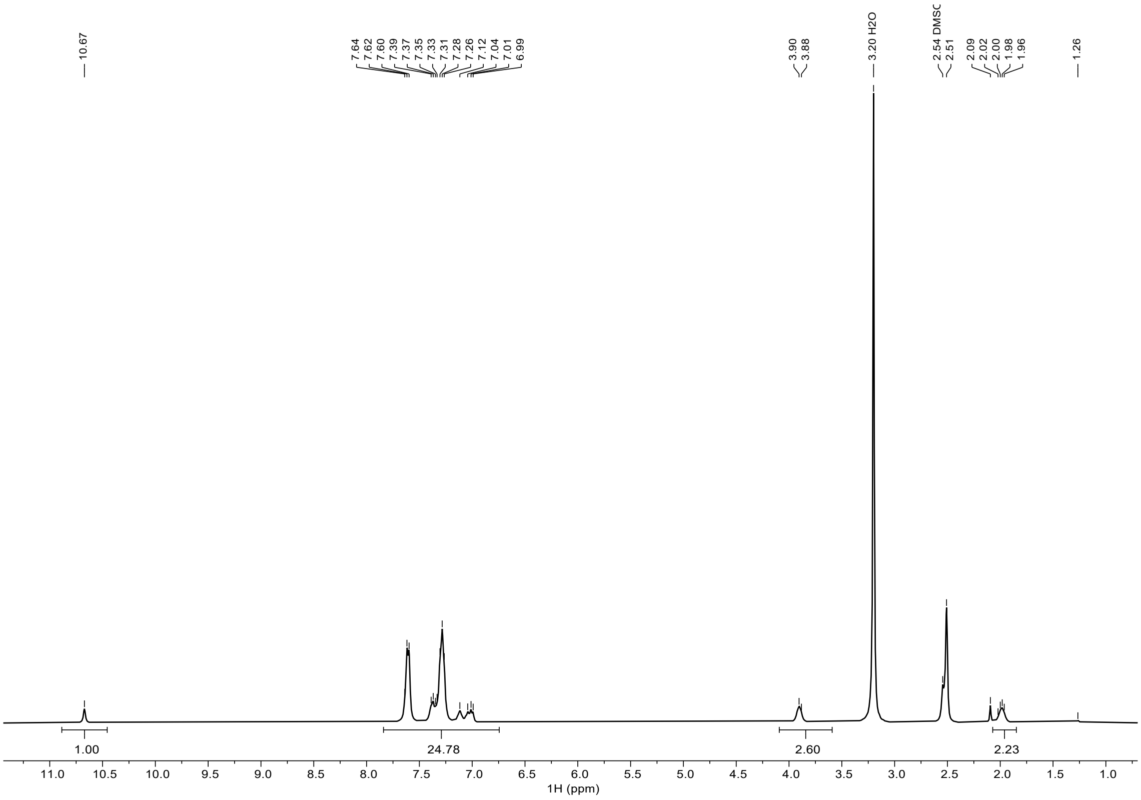


**Figure S4.** $^{1}H$-NMR (400 MHz, DMSO-$d_6$) spectrum of BPIS-65.

### S1.2 Inherent viscosity

The Inherent viscosity ($\eta_{inh}$) of polymers was determined at 30 °C using a Lauda iVisc system and an Ubbelohde viscometer. Polymer solutions with a concentration of 0.5 g · $dL^{-1}$ in NMP were prepared. The inherent viscosity was calculated according to the following equations:

$$\eta_{inh} = \left( \frac{\ln\left(\frac{t}{t_0}\right)}{C} \right) \quad \text{(S1)}$$

Where $t$ is the average flow time of the polymer solution, $t_0$ is the average flow time of the solvent, and $C$ is the concentration of the polymer solution.

**Table S1.** Inherent viscosities of BPIS and BPIS-65. (*) 0.5 % polymer solutions in 1-methyl-2-pyrrolidinone (NMP)

| Sample | $\eta_{inh}$ (g·$dL^{-1}$)* |
|---|---|
| BPIS | 1.76 ± 0.01 |
| BPIS-65 | 3.87 ± 0.01 |

### S1.3. Thermogravimetric analysis (TGA) of BPIS membranes

The effect of N-sulfopropylation of BPIS on the thermal stability of the membranes was studied by thermogravimetric analysis (TGA). Weight Losses between 180 °C and 300 °C were attributed to residual solvent. The degradation onset temperature is listed in Table S2. It was observed that thermal stability of membranes decreased with the amount of $-SO_3^-$ groups. All samples showed char yields at 800 ºC above 70%.

**Table S2.** Degradation temperature and char yield of polymeric membranes.

| Sample | $T_d$ (°C) | Char yield at 800 °C (%) |
|---|---|---|
| BPIS | 488 | 76% |
| BPIS-25 | 474 | 71% |
| BPIS-45 | 471 | 72% |
| BPIS-65 | 455 | 70% |

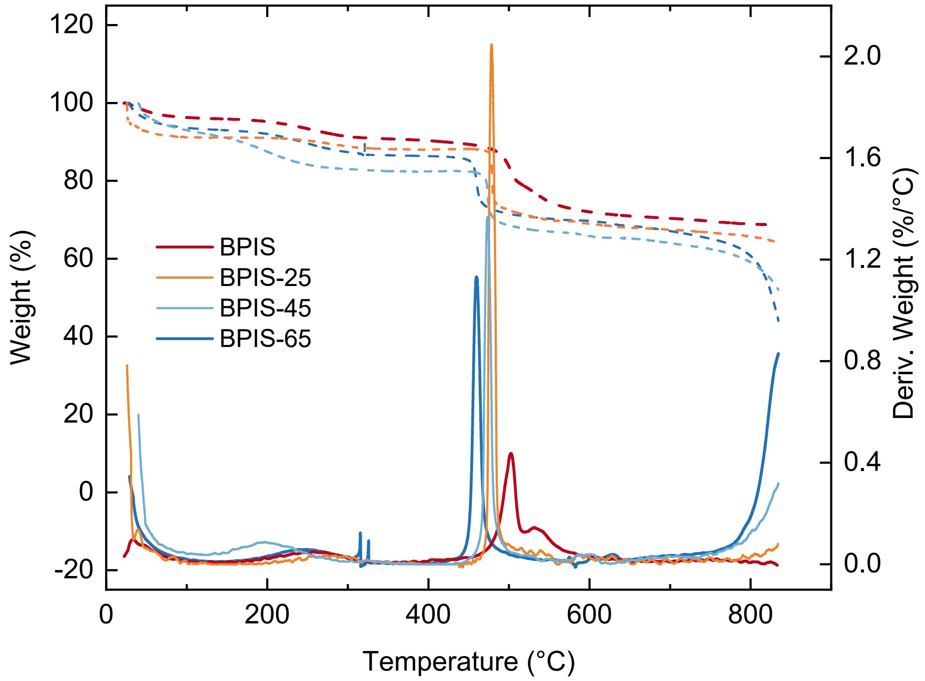


**Figure S5.** Thermogravimetric analysis curves of BPIS, BPIS-25, BPIS-45 and BPIS-65. Polymers with greater SD content showed lower degradation temperature compared with BPIS. All polymers were dried at 120ºC before doing the analysis. Weight loss between 100ºC and 350ºC were attributed to residual solvent and not to the degradation of sulfonic groups. This statement was validated after a post-analysis conducted after subjecting the sample to 350°C.

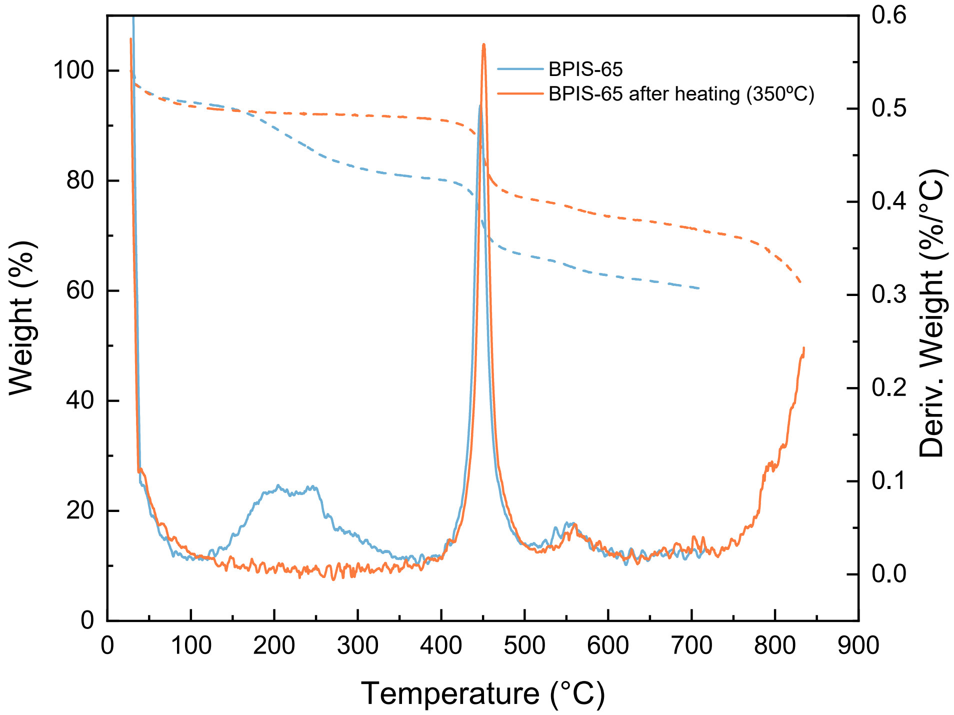


**Figure S6.** Post analysis of BPIS-65 sample to assign weight loss between 120 and 300ºC. Weight loss was assigned to residual solvent in the samples.

### S.1.4. Tensile strength

We carried out uniaxial deformation tests to assess the mechanical strength of the different polymeric membranes. Five samples were examined for each membrane, and the mean values for each property are presented in Table S3.

**Table S3.** Mechanical properties of BPIS membranes: Tensile Strength (TS), Deformation at break (EB), and Young's modulus (E), measured under a tensile load at 5 mm·min$^{-1}$.

| | **TS (MPa)** | **EB (%)** | **E (GPa)** |
|---|---|---|---|
| BPIS | 85.0 ± 13.6 | 9.4 ± 2.0 | 1.93 ± 0.15 |
| BPIS-25 | 59.9 ± 8.1 | 6.5 ± 1.9 | 1.25 ± 0.29 |
| BPIS-45 | 60.1 ± 7.4 | 8.5 ± 2.5 | 1.28 ± 0.32 |
| BPIS-65 | 86.5 ± 2.7 | 11.9 ± 5.0 | 1.26 ± 0.37 |

A comparison of the modulus between membranes shows that the values for sulfonated BPIS are like those of pristine BPIS. This demonstrates their robustness and enables them to be used in compressed RFBs.

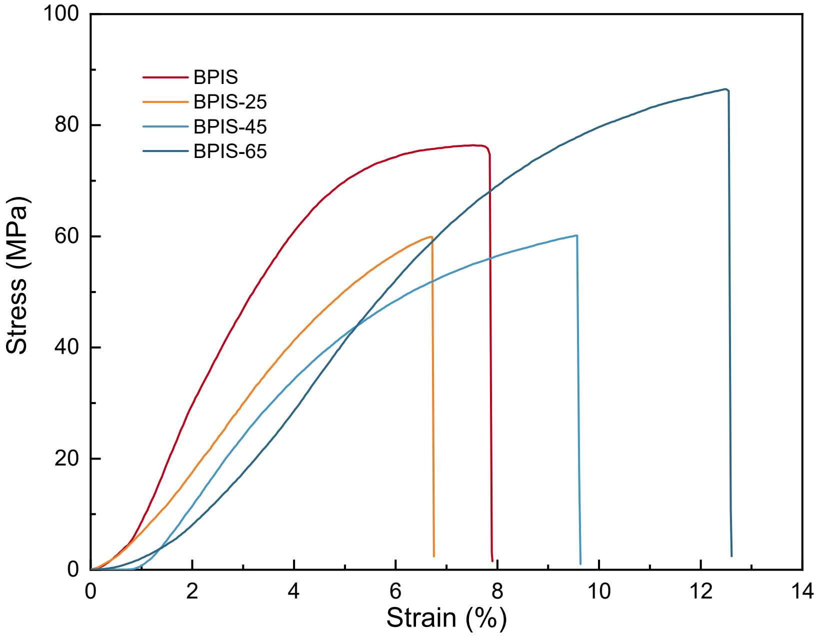


**Figure S7.** Strain-stress curves of BPIS, BPIS-25, BPIS-45 and BPIS-65 samples under a tensile load applied at 5 mm·min$^{-1}$.

### S.1.5. Water Uptake and Swelling Ratio

Water uptake (WU) was calculated by mass changes by Thermogravimetric Analysis (TGA). Thermogravimetric analysis was conducted at 20 °C·min$^{-1}$ using a High-resolution (Hi-Res) mode in a temperature range from 30 °C to 300 °C. The sensitivity and resolution parameters of Hi-Res mode were set to 1 and 4, respectively.

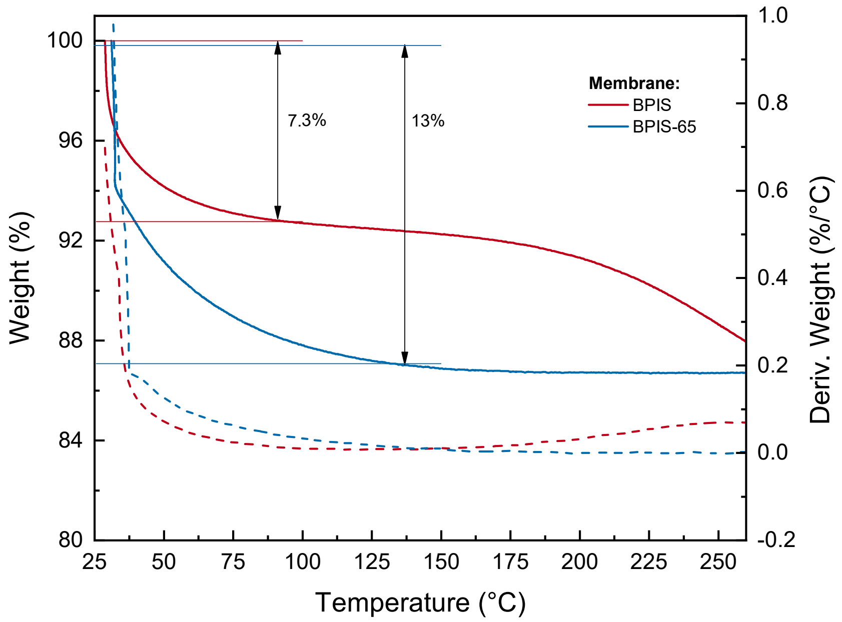


**Figure S8.** Thermogravimetric analysis curves of BPIS and BPIS-65. Membrane with the highest SD content exhibited a higher water uptake compared with BPIS.

As shown in Figure S8, the thermograms of BPIS and BPIS-65 indicate that the water uptake (WU) of the more sulfonated membrane, BPIS-65, is 6% greater than that of BPIS.

The Swelling Ratio (SR) of our membranes was measured by determining the change in the dimensions of the sample (diameter and thickness). Membrane swelling ratio (SR) was calculated according to the following equation:

$$SR\ (\%) = \frac{D_{wet} - D_{dry}}{D_{dry}} \qquad \text{(S2)}$$

where $D_{dry}$ and $D_{wet}$ are the dimensions of the dried and fully hydrated membrane samples, respectively. Diameter changes were observed using optical microscopy and the thickness changes were measured using a coating thickness gauge and are presented in Table S4.

**Table S4.** Swelling Ratio (SR) of BPIS and BPIS-65 membranes. The membrane SR is measured by the change in diameter of membrane samples in dry and wet conditions ($D_{dry}$ and $D_{wet}$).

| | $D_{dry}$ (mm) | $D_{wet}$ (mm) | SR |
|---|---|---|---|
| **BPIS** | 12.7 | 12.8 | 1 % |
| **BPIS-65** | 12.7 | 13.0 | 2 % |

No significant change in dimensions of the samples was observed. This agrees with the low WU of the samples.

### S.1.6. Surface Morphology and Elemental Analysis

The morphologies of BPIS and sulfonated BPIS membranes (BPIS-25, BPIS-45, BPIS-65) were examined using Scanning Electron Microscopy (SEM) (Figure S9 and Figure S10). The membrane surfaces are smooth and uniform, typical of dense membranes.

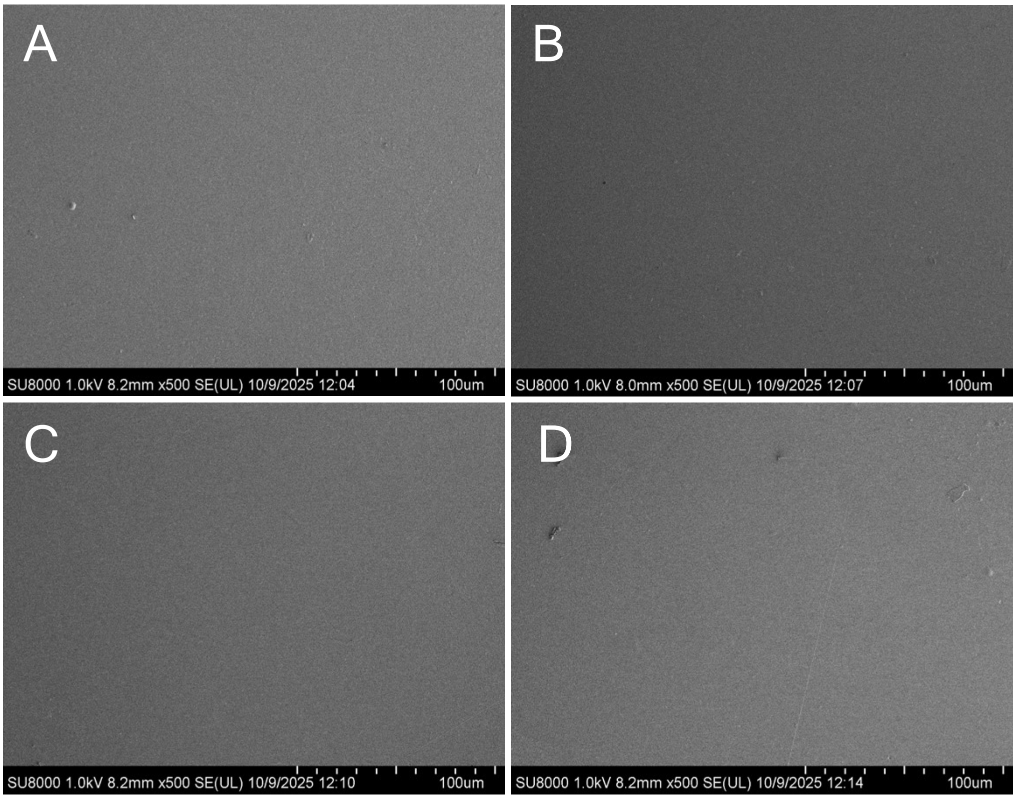


**Figure S9.** SEM-surface images of BPIS (A), BPIS-25 (B), BPIS-45 (C), BPIS-65 (D).

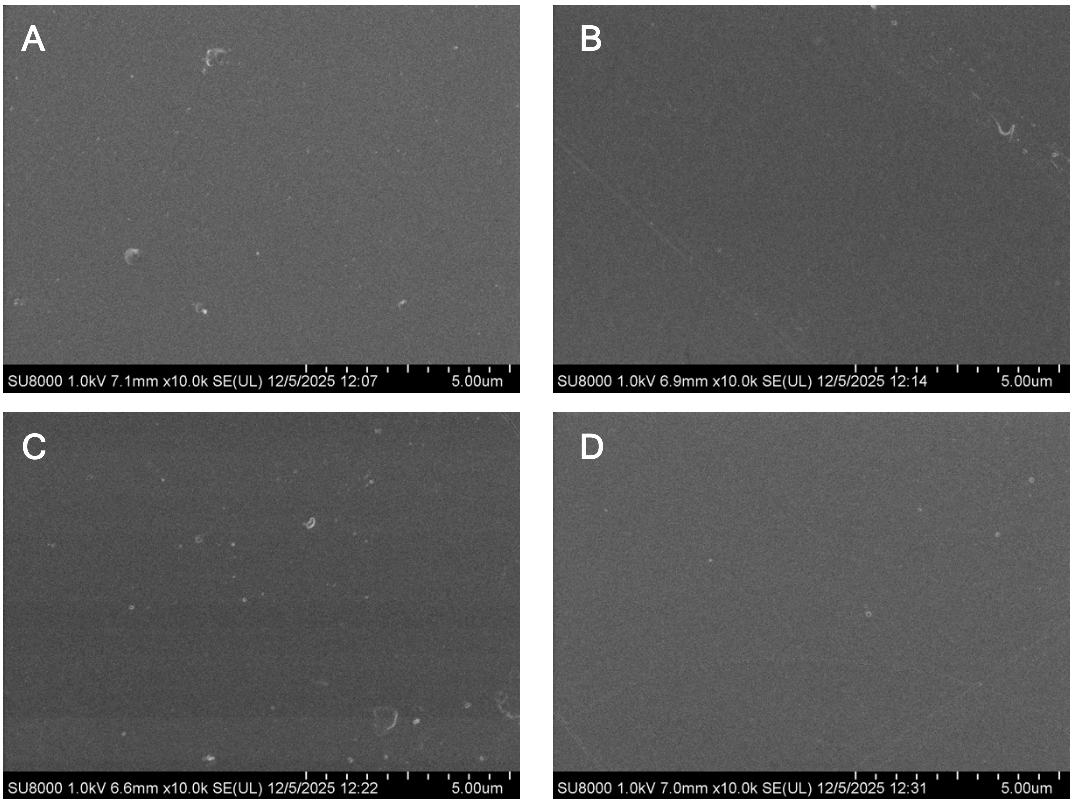


**Figure S10.** SEM-surface images at higher magnification of BPIS (A), BPIS-25 (B), BPIS-45 (C), BPIS-65 (D).

Energy-Dispersive X-ray Spectroscopy (EDX) was used to quantify elemental composition and assess the distribution of sulfonated groups in the modified membranes. The sulfur (S) content increased with the degree of sulfonation in the membranes analyzed, indicating effective polymer functionalization (Table S5). Element distribution maps were generated to examine the uniformity of sulfonic group distribution across the membrane surface (Figure S11-12).

**Table S5.** Elemental Analysis of BPIS membranes obtained by EDX-SEM

| | Elemental Analysis (%) | | | |
|---|---|---|---|---|
| **Membrane** | **C** | **N** | **O** | **S** |
| BPIS | 73.90 | 9.27 | 16.78 | **0.06** |
| BPIS-25 | 70.96 | 5.57 | 16.85 | **4.19** |
| BPIS-45 | 70.20 | 5.39 | 16.84 | **4.62** |
| BPIS-65 | 67.85 | 5.28 | 17.82 | **5.49** |

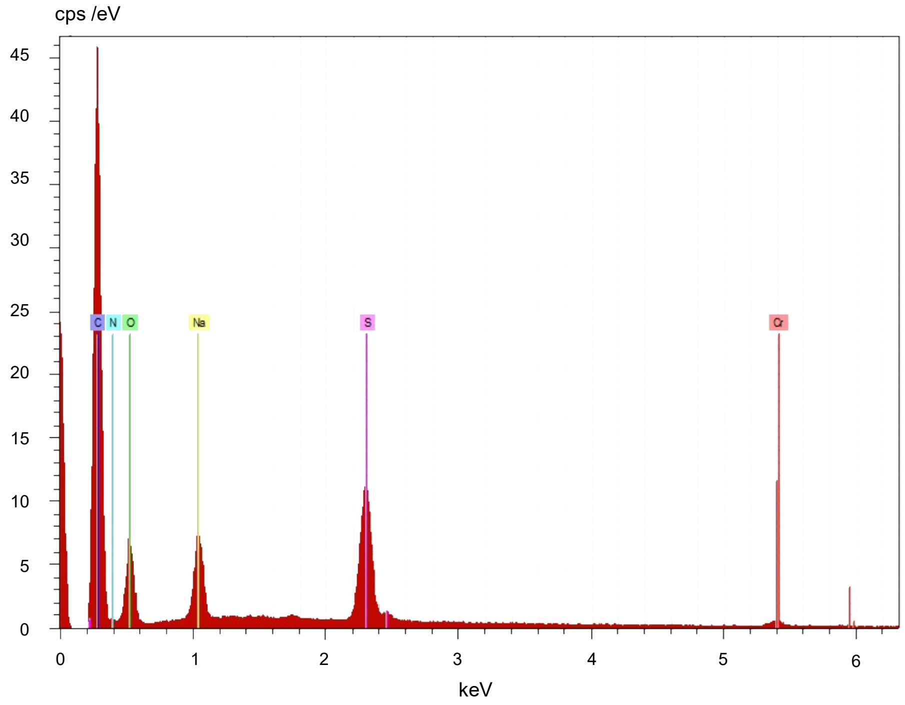


**Figure S11.** EDX elemental analysis results graph of BPIS-65. Chromium content in the sample is due to the metallization process required for the measurements.

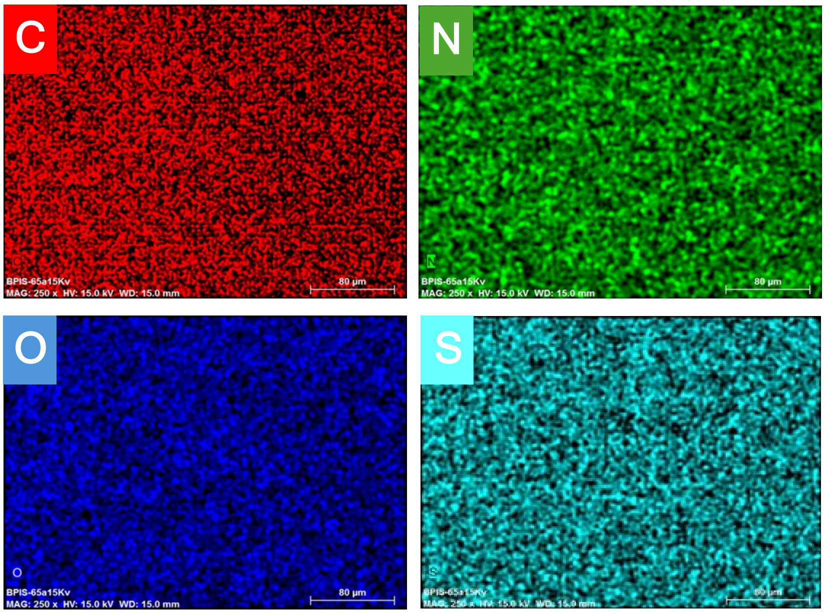


**Figure S12.** EDX elemental distribution maps illustrating the atomic content of BPIS-65: Carbon (red), Nitrogen (green), Oxygen (dark blue), and Sulfur (light blue).

## S.2 Electrochemical Impedance Spectroscopy (EIS)

Ionic conductivity of BPIS membranes in LiCl was measured by electrochemical impedance spectroscopy (EIS). A multichannel potentiostat (Biologic SP-300) was used to perform the tests in a frequency range of 7 MHz – 10 mHz Temperature was controlled with a Buchi glass oven (B-585 model).

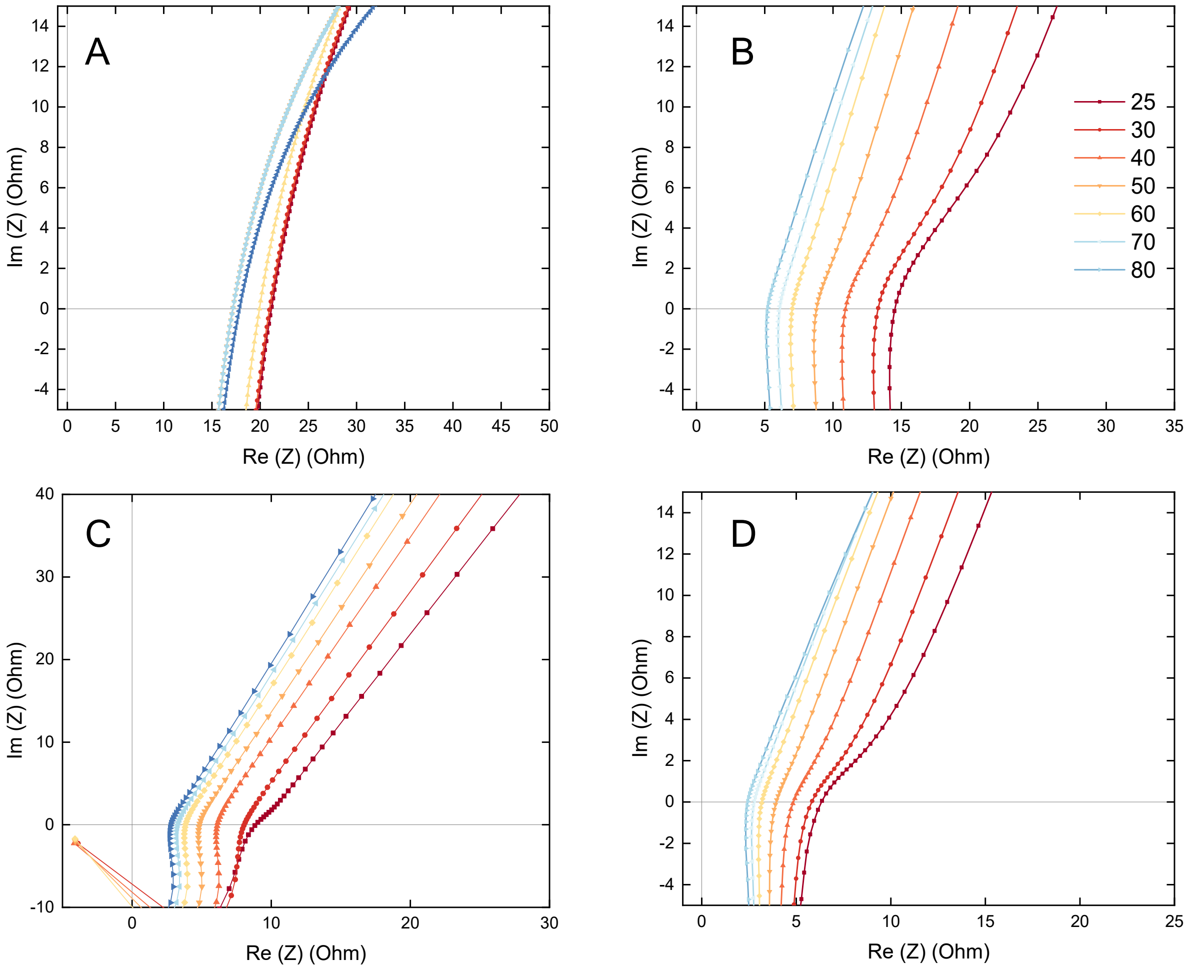


**Figure S13.** Nyquist plots for (A) BPIS, (B) BPIS-25, (C) BPIS-45, and (D) BPIS-65, measured in 1 M aqueous LiCl by EIS in the temperature range of 25-80 °C.

EIS measurements were analyzed using ZFit analysis tool from EC-Lab® software. The results obtained are listed in the table below. The electrical circuit chosen to fit the data is described as follows:

- L1: Inductance represents the electrical resistance of wires and current collectors.
- R1: Resistance of ions to diffuse across the membrane.
- C1: Electrodes and membrane act also as a dielectric element, with delocalized charges in both sides of the membrane.
- Q1: Represents the polarization of the system at lower frequencies.

**Table S6.** Analysis of the Nyquist plots displayed in Figure S12**.**

| Membrane | Equivalent Circuit | $R_i$ (Ω) | $\chi^2/Z^2$ |
|---|---|---|---|
| BPIS-25 | L1+R1/C1+Q1 | 12.75 ± 0.07 | 0.024 |
| BPIS-45 | L1+R1/C1+Q1 | 5.65 ± 0.09 | 0.094 |
| BPIS-65 | L1+R1/C1+Q1 | 7.48 ± 0.05 | 0.039 |
| BPIS | L1+R1/C1+Q1 | 20.3 ± 0.06 | 0.667 |

## S.3 Membrane permeability assessment

In concentration gradient-driven diffusion and crossover experiments with constant volume, the permeation rate of ions and redox molecules across the membrane over a brief period follows Fick's first law.

$$J = \frac{V}{A} \cdot \left(\frac{\partial C}{\partial t}\right) \qquad V \cdot \frac{dc_P}{dt} = J \cdot A \tag{S3}$$

Where $J$ represents the diffusion flux, $V$ ($cm^3$) is the tank volume, $A$ the effective membrane area ($cm^2$), $t$ is time (s), $C$ is the concentration ($mol \cdot cm^{-3}$), and $c_P$ the concentration in the permeate tank ($mol \cdot cm^{-3}$). If we assume linear concentration gradient across the membrane, the permeate flux rate can be defined as:

$$J = \frac{P}{e} \cdot (c_F - c_P) \tag{S4}$$

Where $P$ represents the permeability ($cm^2 \cdot s^{-1}$), $e$ is membrane thickness (cm), and $c_F$ the concentration in the feeding tank ($mol \cdot cm^{-3}$). By substituting the permeation rate in S1, we can obtain a relationship between the evolution of permeate tank concentration over time:

$$V \cdot \frac{dc_P}{dt} = P \cdot \frac{A}{e} \cdot (c_F - c_P) \tag{S5}$$

Assuming that $c_F$ is much larger than $c_P$, $c_F$ can be treated as a constant. The linearization of the system leads to the following equation:

$$\ln\left(1 - \frac{c_P}{c_F}\right) = -\frac{P \cdot A}{V \cdot e} \cdot t \tag{S6}$$

'In order to establish a relationship between ion concentration in solution and conductivity, alibration curves were obtained for each aqueous solution under study (Figure S14).

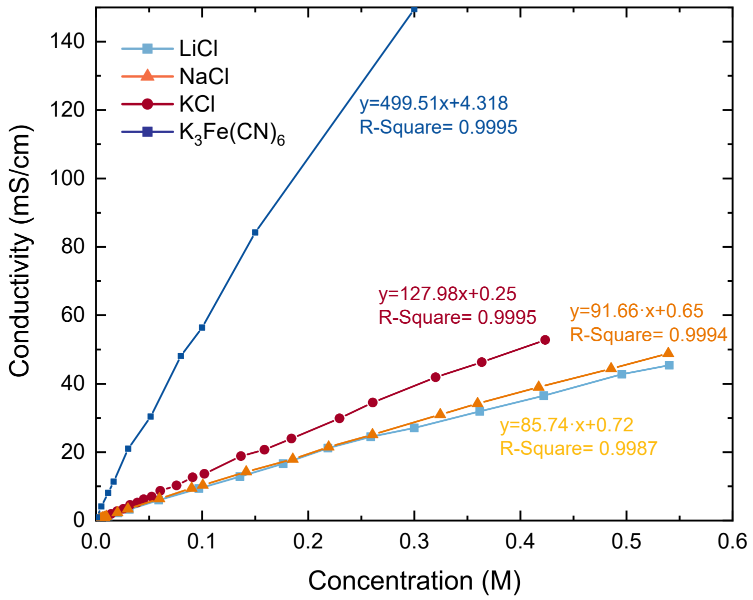

**Figure S14.** Linear regression plots of conductivity as a function of concentration for LiCl, NaCl, KCl and $K_3Fe(CN)_6$ used to calibrate conductivity measurements.

Figure S15 presents the permeability curves of aqueous LiCl solution across the different membranes evaluated. The evolution of LiCl concentration over time reveals active ion permeation, driven by the concentration gradient across the membrane. Each membrane permeability was calculated from the slope of the curves, providing a quantitative measure of ion diffusion in the system.

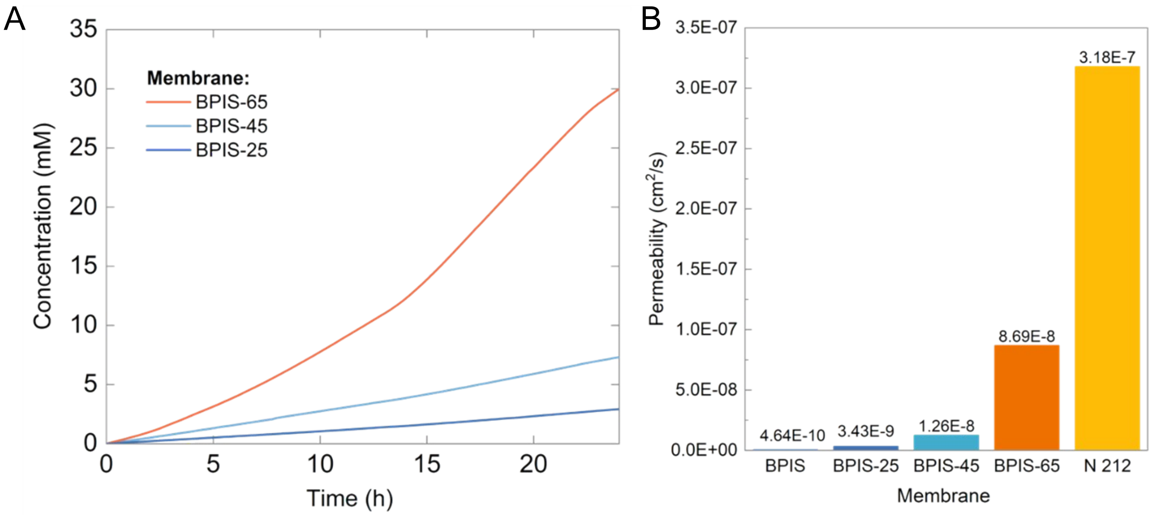


**Figure S15.** (A) Permeate concentration evolution in diffusion test with LiCl 1M as feed solution. (B) LiCl total permeability of the different isatin derived polymeric films compared to Nafion 212. Permeability was calculated by fitting the data to Fick's law.

It is important to note that all permeability experiments were initially conducted using freshly prepared membranes without any pretreatment. To enable a meaningful comparison with commercial membranes, an additional set of permeability tests was performed after subjecting the membranes to a pretreatment process. Specifically, the membranes were soaked for 24 hours in an aqueous LiCl solution, following a similar activation procedure to that used for Nafion membranes. The corresponding results are presented in Table S7.

**Table S7.** Permeability values of BPIS-65 compared with Nafion 212 as the benchmark.

| **Membrane** | LiCl ($cm^2 \cdot s^{-1}$) | $K_3Fe(CN)_6$ ($cm^2 \cdot s^{-1}$) |
|---|---|---|
| BPIS-65 | $8.7 \times 10^{-8}$ | $3.4 \times 10^{-11}$ |
| BPIS-65 (activated) | $1.4 \times 10^{-7}$ | $8.0 \times 10^{-12}$ |
| Nafion 212 (activated) | $3.0 \times 10^{-7}$ | $4.7 \times 10^{-10}$ |

## S.4 In-line operando $^7$Li NMR experiments

### S.4.1 Calibration for Li$^+$ quantification in flow

Quantification of nuclei concentration from an NMR spectrum relies on the principle that NMR signal intensity is directly proportional to the number of resonant nuclei, provided that experimental conditions remain consistent. To calibrate the system, LiCl solutions of known concentrations (0.025, 0.050, 0.100, 0.500, 1.000, and 1.500 M) were prepared and introduced into the magnet at a flow rate of 4 mL·min$^{-1}$. The $^7$Li signal from each spectrum was integrated and plotted against the corresponding LiCl concentration. The resulting linear relationship was subsequently used to determine the concentration of Li$^+$ ions that diffused across the membrane during the inline experiments. The result for the flow calibration is depicted in Figure 6C in the main text.

### S.4.2 Evans method

The general form of the Evans equation is[1]:

$$\Delta\delta = \left(\frac{4\pi}{3} - \alpha\right)\frac{\Delta\chi_m}{V_m} \quad \text{(S7)}$$

where $\Delta\delta$ is the chemical shift difference between an inert compound in the presence and absence of a paramagnetic ion, $\alpha$ is the shape-factor, $\Delta\chi_m$ is the molar magnetic susceptibility, and $V_m$ is the molar volume of paramagnetic species. In our case, the value of $\alpha$ remains unknown due to the undisclosed specifications of the magnet design. As a result, the most practical approach to applying the Evans method, without relying on the full theoretical expression, is to establish an empirical calibration curve. This becomes more evident when the equation is expanded to explicitly relate the chemical shift to the concentration of the paramagnetic species:

$$\Delta\delta = \left(\frac{4\pi}{3} - \alpha\right)\frac{N_A\mu^2}{3K_BT}C_{para} \quad \text{(S8)}$$

where $N_A$ is Avogadro's number, $\mu$ is the effective magnetic moment of the paramagnetic species, $k_B$ is Boltzmann's constant, $T$ is the absolute temperature, and $C_{para}$ is the molar concentration of the paramagnetic ion of interest. All the terms that are constant in this expression can be grouped into a single term, yielding the simplified form:

$$\Delta\delta = constant \cdot C_{para} \quad \text{(S9)}$$

This relationship enables experimental calibration using standard solutions of known paramagnetic species concentrations. To determine this constant, five solutions containing 1M LiCl (as a constant source of $^7$Li nuclei) were prepared with an increasing concentration of $K_3Fe(CN)_6$ (0, 0.025, 0.050, 0.075 and 0.1M). We chose to use the potassium ferric salt instead of the substituted, lithiated one, to have a known constant concentration of Li$^+$ in solution. The solutions were measured with the same acquisition parameters as mentioned before, at a flow rate of 4 ml·min$^{-1}$. The chemical shift of $^7$Li was extracted directly from the processed spectra. To illustrate the evolution of the $^7$Li signal during operando measurements, Figure S16 shows representative spectra extracted at key points throughout the in-line battery experiment (see Figure 5 in the main text).

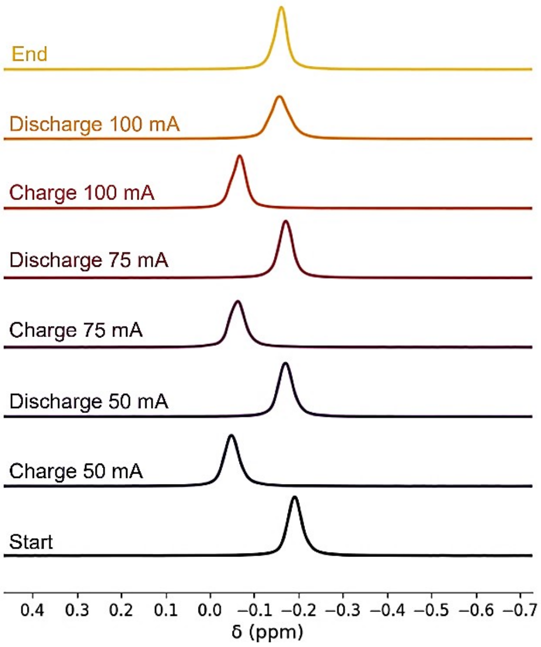


**Figure S16.** Representative $^7$Li-NMR spectra extracted from the *operando* RFB experiment shown in Figure 5 of the main text. The spectra correspond to the electrolyte in the negolyte reservoir at the start of the experiment and after each charge and discharge step at different currents (50, 75, and 100 mA).

A slight broadening of the $^7$Li resonance is observed as the polarity changes between charging and discharging, particularly at higher current densities, consistent with rapid chemical-shift changes during acquisition. This effect, known as 'peak smudging' is modest but may contribute to the reduced apparent $Li^+$ signal at transitional points, although the overall concentration trend and ion balance remain consistent with increased transport of lighter cations under these conditions.

As previously mentioned, the presence of paramagnetic species impacts the transverse relaxation time of nearby nuclei, resulting in broader NMR signals. This line broadening reduces spectral resolution and makes it more difficult to accurately determine the chemical shift. Since the Evans method relies on the precise measurement of the chemical shift difference to quantify $Fe^{3+}$ concentration, increased line broadening can introduce an error in the extracted [$Fe^{3+}$] values. To account for this effect, we estimated the associated uncertainty by measuring the Full Width at Half Maximum (FWHM) of our signal. Similarly, because the $Li^+$ concentration was derived from the $Fe^{3+}$ calibration, we considered that discrepancies in $Li^+$ values might reflect propagated error from both calibrations. To account for this effect, we used the full width at half maximum (FWHM) of the $^7$Li signal as an estimate of the uncertainty in the chemical shift measurement ($\Delta\delta$). Given that the Evans calibration is linear (Equation S9), we estimate the corresponding error in $Fe^{3+}$ concentration using the relation:

$$\sigma_{[Fe^{3+}]} = \frac{0.5 \cdot FWHM}{|constant|} \tag{S10}$$

Where $\sigma_{[Fe^{3+}]}$ is the uncertainty associated with the $Fe^{3+}$ concentration, and *constant* is the slope in Figure 6. The factor of 0.5 reflects the assumption that the maximum deviation of the peak's center is half the linewidth.

### S.4.3 Associated uncertainty in $Li^+$ quantification method

We also aimed to quantify the uncertainty associated with the extracted $Li^+$ concentration. We assume that the predominant source of uncertainty is associated with the measured signal intensity ($I_{apparent}$). The standard deviation of this intensity ($\sigma_{apparent}$) was taken from the baseline noise level of the NMR spectra. Then, from our exponential calibration model:

$$I_{corr} = 0.2\ (5.7 - e^{-[Fe^{3+}]/34.9\ mM}) \quad \text{(S11)}$$

The uncertainty on $I_{corr}$ arises both from uncertainty in $Fe^{3+}$ concentration and from the calibration curve parameters. Its uncertainty can be defined as:

$$\sigma_{corr} = \left|\frac{dI_{corr}}{d[Fe^{3+}]}\right| \cdot \sigma_{[Fe^{3+}]} \quad \text{(S12)}$$

Finally, knowing the uncertainties associated, the standard error propagation for a ratio of two quantities:

$$\sigma_{real} = I_{real} \cdot \sqrt{\left(\frac{\sigma_{apparent}}{(I_{apparent})}\right)^2 + \left(\frac{\sigma_{corr}}{I_{corr}}\right)^2} \quad \text{(S13)}$$

Gives the total propagated uncertainty in the corrected $Li^+$ signal intensity.

### S.4.4 Validation of $Li^+$ quantification methodology

To validate our approach for correcting SoC-dependent and flow-dependent changes in the $^7Li$ signal, we performed an additional inline experiment under identical battery cycling conditions. This time the solutions were continuously flowed for 10 min following completion of the charge process, after which the flow was halted while the NMR acquisition continued (Figure S17). This approach allows direct comparison between (i) concentrations obtained under flow using the relaxation-corrected signal intensities and (ii) concentrations obtained under static conditions using direct signal integration and a static calibration. All acquisition parameters were kept identical. The $Li^+$ concentration under static conditions was obtained directly from the integrated $^7Li$ signal using the calibration in Figure S18, as no flow-induced attenuation is present. As shown in Table S8, this value is in good agreement with the $Li^+$ concentration obtained under flow using our correction method. This confirms that our procedure reliably compensates for both SoC-dependent changes in relaxation and flow-induced signal attenuation.

**Table S8.** Comparison of $Li^+$ concentrations obtained under flow (4 $mL{\cdot}min^{-1}$) and stop-flow (0 $mL{\cdot}min^{-1}$) conditions at a current density of 15 $mA{\cdot}cm^{-2}$. The consumed $Fe^{3+}$ was quantified using the Evans method. Under flow conditions, the charge-balancing $Li^+$ concentration was determined using the corrected calibration procedure and the flow calibration in Figure 6C; whereas the stopped-flow concentration was obtained directly from the integrated $^7Li$ NMR signal, following the calibration provided in Figure S18.

| Region | Time point | Flow Rate | Consumed $[Fe^{3+}]$ | $^7Li$ Intensity (A.U.) | Balancing $[Li^+]$ |
|---|---|---|---|---|---|
| 1* | 22 min | 4 $mL{\cdot}min^{-1}$ | 35.89 ± 0.01 mM | $3.58 \cdot 10^9$ | 36.2 mM |
| | 23 min | | 35.89 ± 0.01 mM | $3.58 \cdot 10^9$ | 36.2 mM |
| | 24 min | | 35.89 ± 0.01 mM | $3.58 \cdot 10^9$ | 36.2 mM |
| 2* | 29 min | 0 $mL{\cdot}min^{-1}$ | 35.89 ± 0.01 mM | $5.66 \cdot 10^9$ | 36.3 mM |
| | 30 min | | 35.89 ± 0.01 mM | $5.66 \cdot 10^9$ | 36.4 mM |

| | | | | | |
|---|---|---|---|---|---|
| | 32 min | | 35.89 ± 0.01 mM | 5.66 · $10^9$ | 36.3 mM |

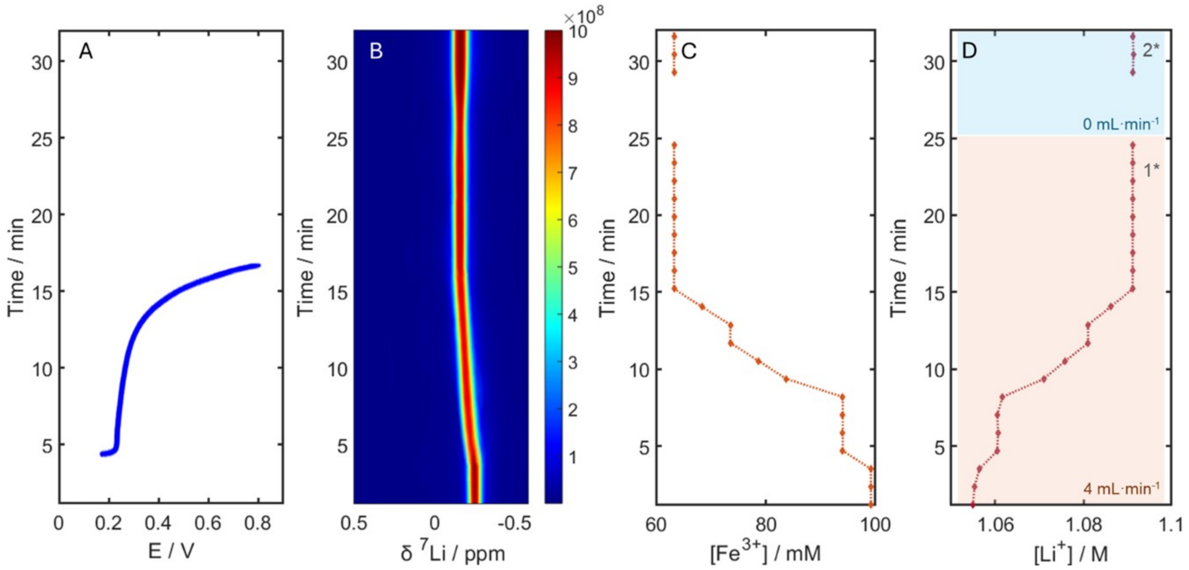


**Figure S17.** (A) Voltage profile of a symmetric cell containing 100 mM $Li_3Fe(CN)_6$ against 100 mM $Li_4Fe(CN)_6$, obtained at a current density of 15 mA·cm$^{-2}$. (B) Inline pseudo-2D $^7$Li spectra of $Li_3Fe(CN)_6$ reservoir. The discontinuity between the flow and static regimes corresponds to the time required for temperatures of the benchtop NMR shim coil and probe to stabilize after the rapid change of flow rate in the detection region, this manifests as a gradual increase in signal intensity until stable temperatures are re-established. (C) Evans method-derived $Fe^{3+}$ concentration. (D) $Li^+$ concentration extracted using our relaxation correction method (4 mL·min$^{-1}$) and measured directly under stopped flow (0 mL·min$^{-1}$).

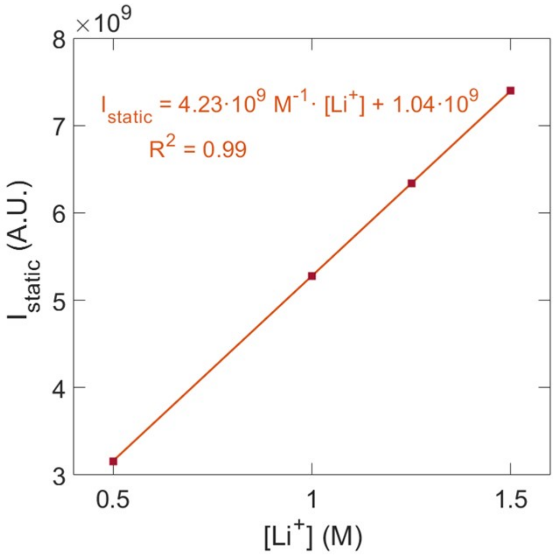


**Figure S18.** Linear fitting between the integrated signal intensity of the calibrate solutions with various LiCl concentrations under stop flow conditions. The $R^2$ of the fitting is 0.99.

For clarity, here we explicitly show how the $Li^+$ concentration under flow was calculated.

Taking the first row of Table S8, the information we get from the experiment is as such:

| Time (min) | $\Delta\delta$ (ppm) | $[Fe^{3+}]$ (mM) | $I_{apparent}$ (A.U.) | $I_{corr}$ | $I_{real}$ (A.U.) | $[Li^+]$ (M) |
|---|---|---|---|---|---|---|
| 22 | -0.15 | 63.28 | 3.584 · $10^9$ | 1.126 | 3.183 · $10^9$ | 1.091 |

Where $\Delta\delta$ and $I_{apparent}$ were extracted from the processed spectra directly. Having these two values, we can then calculate [$Fe^{3+}$] from the Evans method (Figure 6.A):

$$\Delta\delta = -2.41\, M^{-1} \cdot [Fe^{3+}]$$

Now, using the empirical relationship extracted from Figure 6B:

$$I_{corr} = 0.20\,(5.79 - e^{-\frac{[\mathrm{Fe}^{3+}]}{34.43\, mM}})$$

Substituting [$Fe^{3+}$] results in a correction factor $I_{corr} = 1.126$.
Using Equation (2):

$$I_{real} = I_{apparent} \cdot I_{corr}^{-1} = 3.183 \cdot 10^{9}$$

Finally, [$Li^{+}$] was obtained by applying the calibration in Figure 6C:

$$I_{real} = 2.90 \cdot 10^{9}\, M^{-1} \cdot [Li^{+}] + 1.84 \cdot 10^{7}$$

Thus,

$$[Li^{+}] = 1.091\, M$$

For calculation in static conditions, meaning no flow, the integrated intensity can be directly applied to the calibration shown in Figure S18:

$$I_{static} = 4.23 \cdot 10^{9} M^{-1} \cdot [Li^{+}] + 1.04 \cdot 10^{9}$$

Considering the last row shown in Table S8, where $I_{static} = 5.655 \cdot 10^{9}$, then:

$$[Li^{+}] = 1.091\, M$$

For each data point in Figure S17, the concentration of charge-balancing $Li^{+}$ was determined from the change in $Li^{+}$ concentration relative to the initial state ($t_0$) :

$$\Delta[Li^{+}]\,(Balancing\ ions) = [Li^{+}]_{t} - [Li^{+}]_{t0}$$

Using an initial $Li^{+}$ concentration of 1.055 M, both the flow-corrected and stopped-flow measurements yield a net $Li^{+}$ transport of approximately 36 mM, confirming consistency between the two approaches.

## S.5 In-line conductivity measurement of the BPIS-65 half cell

To investigate how the membrane behaves under more demanding conditions, we conducted an additional set of experiments using a flow rate of 40 mL · $min^{-1}$ (Figure S19). During these tests, the conductivity of the external reservoirs was continuously tracked with a conductivity probe (Eutech PC 2700, Thermo Scientific). In-line measurements from the negolyte reservoir showed conductivity variations during battery charging and discharging.

During the charge, initial $Fe^{3+}$ (139.4 mS·$cm^{-1}$) is reduced to $Fe^{2+}$. At the end of the charge cycle, anolyte tank conductivity decreases to 137 mS·$cm^{-1}$ (Figure S19A). This difference is related to the conductivity of both redox active salts. At first, the conductivity would be expected to increase in the anolyte tank due to the migration of $Li^+$ from the positive side of the battery to uphold electroneutrality. However, as we have proposed, at high supporting salt concentration, both co-ions and counter ions diffuse through the polymeric membrane. Consequently, these changes observed (Figure S19A) can be attributed to the active material, providing insight into the oxidation or reduction state of the negolyte or catholyte and the state of charge of the battery. The same sequence was followed (Figure S19B) as in the NMR tests (Figure 5B), cycling at three different current regimes (50, 75 and 100 mA).

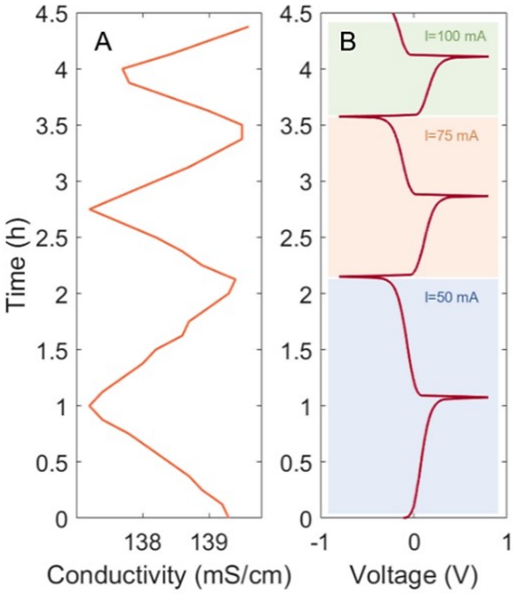


**Figure S19.** (A) Conductivity evolution of negolyte tank ($Li_3Fe(CN)_6$). (B) Voltage profile at three different currents (50, 75, 100 mA).

## S.6 Cycling performance of BPIS-65 symmetric cell

Galvanostatic cycling with BPIS-65 membrane was performed with a 0.1 M $Li_4Fe(CN)_6$ and $Li_3Fe(CN)_6$ aqueous solution, using 1 M LiCl as a supporting salt. Figure S20 displays capacity versus voltage profiles obtained during longer cycling. The graph shows a noticeable capacity loss at cycle 10 due to increased current density, but this loss is recovered when the initial current density (20 mA · $cm^{-2}$) is restored. The half-cell experiment with this membrane enabled successful cycling for over 150 cycles. In addition to the physicochemical characterization of the membrane, it is important to also evaluate performance metrics to gain a comprehensive understanding of the system's electrochemical behavior. Electrochemical impedance spectroscopy (EIS) and voltage polarization measurements were carried out to directly compare the BPIS-65 membrane with the commercial benchmark Nafion 212 (N212).

Galvanostatic cycling experiments with N212 were performed under the same conditions as those used for BPIS-65. The polarization behaviour of BPIS-65 and N212 was evaluated by monitoring the average cell voltage during cycling (Figure S21A). Both membranes exhibited similar polarization profiles, indicating comparable electrochemical performance under identical operando conditions. In contrast, EIS measurements performed under operando conditions showed that BPIS-65 had a significantly lower total resistance than N212 (Figure S21B), suggesting improved ionic conductivity and higher cell efficiency.

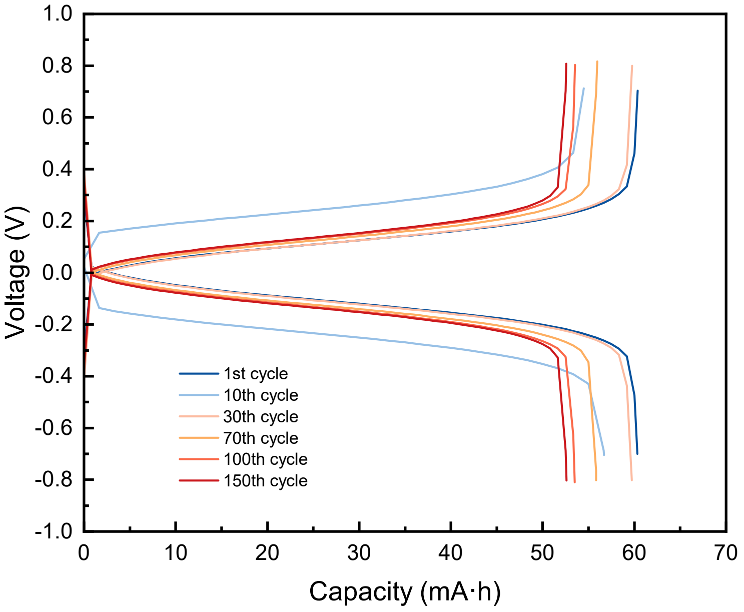


**Figure S20.** Capacity-voltage curves for long cycling experiments with the BPIS-65 membrane.

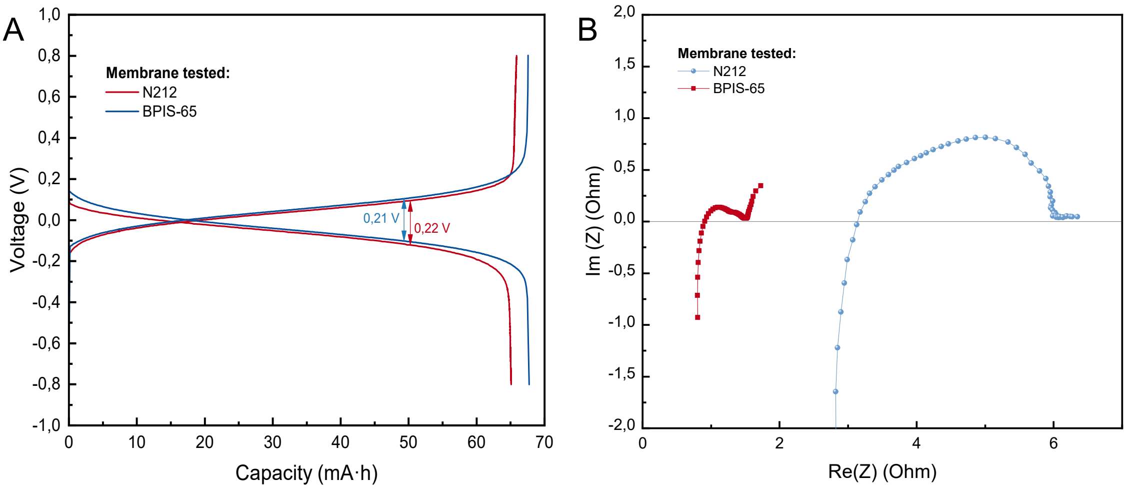


**Figure S21.** (A) Comparison of the capacity–voltage profiles during the 2nd charge–discharge cycle for Nafion 212 and BPIS-65 membranes at 100 mA·cm$^{-2}$. (B) Comparison of EIS spectra for symmetric cells containing Nafion 212 and BPIS-65 membranes with 0.1M $Li_4Fe(CN)_6$ | 0.1 M $Li_3Fe(CN)_6$ electrolytes.

We used EIS to monitor changes in membrane resistance throughout cycling under galvanostatic control. For that purpose, we established the following sequence:

1. EIS measurement.
2. Galvanostatic cycling at 20 mA·cm$^{-2}$.

3. EIS measurement.
4. Galvanostatic cycling for 60 cycles (EIS measurement after 4$^{th}$, 20$^{th}$, 40$^{th}$ and 60$^{th}$ cycle).
5. Galvanostatic cycling at 40 mA·cm$^{-2}$ for 10 cycles.
6. EIS measurement.
7. Galvanostatic cycling at 20 mA·cm$^{-2}$ for 20 cycles.
8. EIS measurement.

Figure S22 illustrates the evolution of impedance with both cycling time and current density. The Nyquist plots reveal a gradual increase in membrane resistance over repeated cycles. Changes in current density also affect the EIS response, with resistance rising more sharply at higher current densities. When the current is returned to its initial value (20 mA·cm$^{-2}$), the resistance does not fully recover, indicating irreversible losses within the system. These irreversible changes likely contribute to the observed capacity fade caused by polarization.

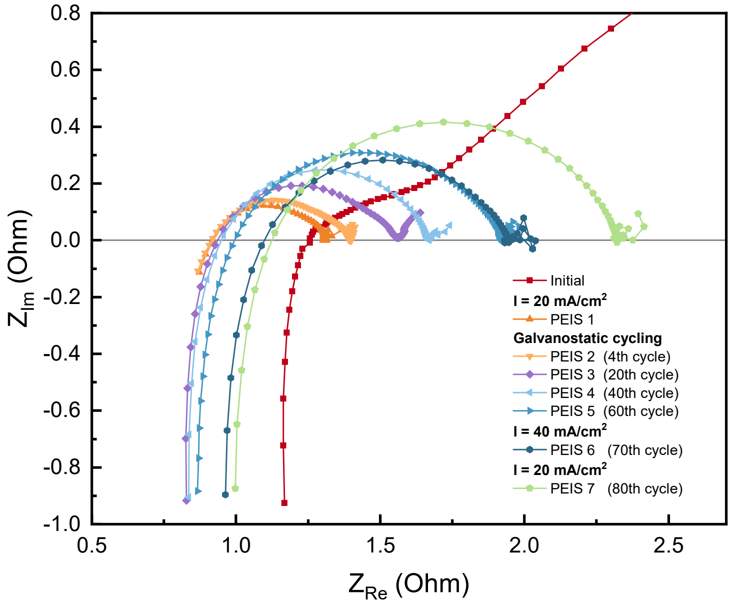


**Figure S22.** EIS spectra of BPIS-65 membrane at different energy densities (10 and 20 mA·cm$^{-2}$) during galvanostatic charge and discharge in a symmetrical RFB 0.1 M $Li_4Fe(CN)_6$ | 0.1 M $Li_3Fe(CN)_6$.

To calculate capacity decay rate over cycling, discharge capacity versus cycles was represented, in order to compare N212 membrane and BPIS-65 membrane (Figure 8C). Both cells were measured under galvanostatic cycling conditions with a current density of 20 mA·cm$^{-2}$ and a flow rate of 50 mL·min$^{-1}$.

To evaluate long-term performance, it is necessary to quantify the capacity decay rate at over time. This parameter offers an easy way to compare the performance behaviour of different membranes in a battery cell. In this study, we have calculated the daily capacity decay rate using the following formula:

$$Decay\ rate\left(\frac{\%}{day}\right) = \frac{C_0 - C_n}{C_n} \cdot \frac{100}{n} \quad \text{(S14)}$$

Where $C_0$ and $C_n$ represent the discharge capacity at first cycle and last cycle respectively and n is the total number of days.
